# Antimony oxide buffer layer for single- and double-junction perovskite-based solar cells

**DOI:** 10.1038/s41467-026-70848-8

**Published:** 2026-03-25

**Authors:** Biao Shi, Zetong Sunli, Pengfei Liu, Wei Han, Rui Kong, Cong Sun, Ying Liu, Yuan Luo, XianZhao Wang, Zhi Zhang, Dekun Zhang, Xiaona Du, Fu Zhang, Miao Yang, Yongcai He, Bo He, Xixiang Xu, Rui Xia, Xueling Zhang, Yifeng Chen, Jifan Gao, Fuzong Xu, Ying Zhao, Stefaan De Wolf, Xiaodan Zhang

**Affiliations:** 1 Institute of Photoelectronic Thin Film Devices and Technology, Renewable Energy Conversion and Storage Center, State Key Laboratory of Photovoltaic Materials and Cells, Nankai University, Tianjin, P. R. China; 2 Tianjin Key Laboratory of Efficient Utilization of Solar Energy, Tianjin, P. R. China; 3 Haihe Laboratory of Sustainable Chemical Transformations, Tianjin, P. R. China; 4 Engineering Research Center of Thin Film Photoelectronic Technology of Ministry of Education, Tianjin, P. R. China; 5 Collaborative Innovation Center of Chemical Science and Engineering (Tianjin), Tianjin, P. R. China; 6 LONGi Central R&D Institute, LONGi Green Energy Technology Co. Ltd., Xi’an, P. R. China; 7 State Key Laboratory of PV Science and Technology, Trina Solar, Changzhou, P. R. China; 8 King Abdullah University of Science and Technology (KAUST), Physical Sciences and Engineering Division (PSE), Material Science and Engineering Program (MSE), Thuwal, Kingdom of Saudi Arabia

**Keywords:** Solar cells, Solar cells

## Abstract

Atomic layer-deposited tin oxide serves as an effective buffer layer in perovskite/silicon tandem solar cells due to its efficient charge extraction and sputtering tolerance. Nevertheless, its unavoidable chemical erosion effect of atomic layer-deposited tin oxide on perovskite requires thicker fullerene charge transport layers, leading to increased parasitic optical absorption. Herein, we firstly integrated thermal evaporated antimony oxide into solar cells to effectively replace atomic layer-deposited tin oxide, enabling a thinner fullerene to minimize optical losses and prevent damage to the perovskite. The unique amorphous-nanocrystalline structure of, antimony oxide facilitates ultrafast carrier transport via its embedded nanocrystalline network. The antimony oxide-based tandem solar cells demonstrated a significant improvement in power conversion efficiency compared to tin oxide-based devices, primarily due to an enhanced short-circuit current density of approximately 1 mA/cm² in the perovskite top cell. Remarkably, even at 64.64 cm^2^ scale, the antimony oxide-based encapsulated large-area tandem solar cell retains an efficiency of 28.16% (with a certified value of 27.70%), attesting the scalability of this approach.

## Abstract (web summary)

Perovskite–silicon solar cells rely on buffer layers that avoid damaging the perovskite, but common tin-oxide layers force thicker transport layers that increase optical loss. The authors replace this layer with thermally evaporated antimony oxide, enabling thinner C_60_ and higher-efficiency perovskite–silicon solar cells.

## Introduction

Monolithic perovskite/silicon tandem solar cells (PSTs) have emerged as highly promising photovoltaic devices, demonstrating exceptional power conversion efficiency (PCE) with a certified value of 34.85%^1^. Nevertheless, the considerable gap relative to the 42% theoretical limit points to critical unresolved losses in optical absorption and charge carrier transport^2^. The engineered functional layers in light-facing front contact architectures, featuring controlled nanoscale thickness^3^, present significant potential for improving photovoltaic performance through enhanced short-circuit current density (*J*_SC_).

Currently, thermal evaporated (TE) fullerene (C_60_) and atomic layer-deposited (ALD) tin oxide (SnO_X_) films are widely used as electron transport layer (ETL) and buffer layer, respectively, in nearly all p-i-n structured PSTs^4–13^. However, the intrinsic ligand exchange reaction between tetrakis(dimethylamino)tin(IV) (TDMASn) precursor in ALD and formamidinium (FA^+^) in the perovskite is one of the main sources of the collapse of perovskite structure^14^. Therefore, it is essential to enhance the thickness and mechanical strength of C_60_ layer to avoid adverse erosion dominated by ALD-SnO_X_. However, this exacerbates the parasitic optical absorption at 300–550 nm wavelength which is a harbinger of the current loss in PSTs^15,16^. Besides, the additional ALD technology further complicates the tandem solar cell fabrication equipment. Thus, it is urgently desirable to develop a gentler technology to prepare the buffer layer to overcome the drawbacks of state-of-the-art SnO_X_.

Herein, we firstly developed TE-antimony oxide (Sb_2_O_3_) film as an innovative alternative to ALD-SnO_X_ for buffer layer applications. Through comprehensive characterization, we identified an amorphous-nanocrystalline hybrid structure in the Sb_2_O_3_ film and elucidated its unique electron transport mechanism, mediated by percolating nanocrystalline channels. Compared to ALD-SnO_X_, TE-Sb_2_O_3_ demonstrates superior process window and minimized adverse effects on perovskite materials. This enhanced compatibility enables the reduction of the C_60_ layer thickness from 15 to 5 nm without compromising perovskite device performance. Consequently, the C_60_/Sb_2_O_3_ stacked functional layer achieves both excellent carrier extraction efficiency and minimized optical parasitic absorption losses. We employed a vacuum-solution hybrid deposition method to fabricate perovskite films with tunable bandgaps (1.59, 1.62, 1.64, and 1.68 eV) and integrated an ultra-thin C_60_/Sb_2_O_3_ film into perovskite solar cells (PSCs), demonstrating universal bandgap applicability. The 1.64 eV-bandgap PSCs achieved a champion PCE of 23.18%, comparable to that of SnO_X_-based PSCs (23.17%). Notably, the 23.18% PCE achieved here represents the highest reported value for mid-/wide-bandgap PSCs fabricated using a vacuum-solution hybrid method. Furthermore, Sb_2_O_3_-based PSTs exhibited an enhanced PCE of 30.28% with an aperture area of 1.0 cm², compared to the 28.59% efficiency of SnO_X_-based PSTs, primarily due to a *J*_SC_ improvement of ~1 mA/cm². The scalability of this approach was successfully demonstrated through the fabrication of 64.64 cm² encapsulated PSTs, which achieved a certified PCE of 27.70%—ranking among the highest reported efficiencies for larger-area PSTs ( > 10 cm²) in the literature.

## Results

### Antimony oxide thin film

**Fig. 1:**
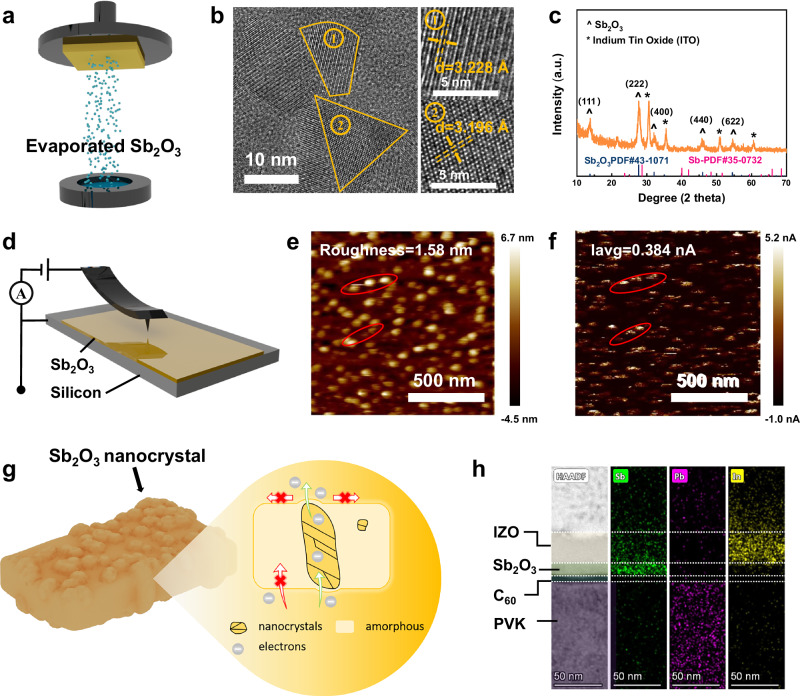
Characterization of Sb_2_O_3_ films. **a** Schematic illustration of the preparation of Sb_2_O_3_ by thermal evaporation method. **b** TEM image of the Sb_2_O_3_ film. **c** XRD spectrum of the Sb_2_O_3_ film. **d** Longitudinal c-AFM measurement setup. AFM (e) and corresponding c-AFM (f) images of the Sb_2_O_3_ film under a bias voltage of 3 V. g, Schematic illustration of conductive mechanism of the Sb_2_O_3_ film. h, Cross-sectional TEM image and the corresponding EDS mapping of PVK/C_60_/Sb_2_O_3_/IZO stack films.

Materials located at the junction of metallic and non-metallic elements in the periodic table are typically used as functional layers in semiconductor devices. N-type metal oxides, e.g. SnO_X_ and titanium oxide (TiO_X_)^17^, have been widely applied in perovskite-based tandem solar cells. However, the high melting points of most metal oxides complicate their preparation via TE (Supplementary Table 1). In contrast, Sb_2_O_3_ is an n-type semiconductor with a low sublimation temperature ( ~ 490 °C under ambient pressure), owing to its molecular crystal structure^18^. Given this unique advantage, we proposed to prepare Sb_2_O_3_ film with evaporation method, as shown in Fig. 1a.

Atomic force microscope (AFM) image based on Sb_2_O_3_ shows a flat and homogenous morphology at the micrometer scale, without discernible voids or bumps (Supplementary Fig. 1). The morphology of 15 nm Sb_2_O_3_ coated on perovskite (PVK)/C_60_ multilayer further validates its homogeneity and flatness by scanning electron microscope (SEM) and AFM (Supplementary Figs. 2, 3). However, only 5 nm Sb_2_O_3_ fails to fully cover the substrate (Supplementary Fig. 3). Transmission electron microscope (TEM) and X-ray diffraction (XRD) measurements indicate that the Sb_2_O_3_ forms an amorphous-nanocrystalline film^19,20^ (Fig. 1b, c, Supplementary Fig. 4). The nanocrystalline structure was identified as cubic with an interplanar spacing of *d* = 3.2 Å. The XRD data further reveals that the crystallites are preferentially oriented with the (222) planes parallel to the glass substrate^21,22^.

To investigate the vertical conductivity of Sb_2_O_3_, we performed conductive atomic force microscopy (c-AFM), applying a 3 V bias to the longitudinal side of silicon/Sb_2_O_3_^23^ (Fig. 1d–f). The c-AFM mapping clearly distinguishes between amorphous and nanocrystalline regions in the Sb_2_O_3_ layer (Fig. 1e). Our findings demonstrate that the nanocrystalline regions enable vertical conduction, whereas the amorphous regions remain insulation (Fig. 1f). Complementary lateral c-AFM measurements further reveal poor lateral conductivity in Sb_2_O_3_ in Supplementary Fig. 5. These results suggest that the nanocrystals can serve as conductive channels for longitudinal electrical transport, making the Sb_2_O_3_ potentially suitable as a buffer layer (Fig. 1g). However, as a wide-bandgap material ( ~ 4.25 eV), Sb_2_O_3_ tends to form high energy barriers at interface (Supplementary Fig. 6). We found that the presence of Sb-interstitial-induced defects generates a quasi-continuum of gap states in Sb_2_O_3_ nanocrystalline (Supplementary Notes 1, 2 and Supplementary Fig. 7-13). These states form a conductive pathway across the interface, allowing electrons to traverse the barrier via defect-mediated transport.

Cross-sectional TEM of the PVK/C_60_/Sb_2_O_3_/indium zinc oxide (IZO) structure was performed to investigate the interlayer behavior of Sb_2_O_3_ (Fig. 1h and Supplementary Figs. 14, 15). A uniform 15 nm Sb_2_O_3_ film exhibits favorable interfacial contact with the amorphous C_60_, owing to their low mutual chemical reactivity and residual stress (Supplementary Notes 3 and Supplementary Figs. 15–17). Notably, energy dispersive spectrometer (EDS) mapping confirms a well-defined layer structure and reveals negligible indium diffusion into the underlying layers, displaying the qualified sputtering tolerance of the Sb_2_O_3_ film (Fig. 1h and Supplementary Fig. 14). As shown in Supplementary Fig. 15, the nanocrystalline also exhibits a cubic structure, aligning with the planar TEM and XRD results (Fig. 1b, c). The structural stability also indicates its excellent sputtering tolerance and simple physic contact style with C_60_ layer via van der Waals forces. Furthermore, we conducted dark photocurrent density-voltage (*J*–*V*) tests with device structure of ITO/Me-4PACz/PVK/C_60_/with or without Sb_2_O_3_/IZO/Al (Supplementary Fig. 18). The Sb_2_O_3_ sample exhibits weaker leakage current, proving the excellent sputtering tolerance of Sb_2_O_3_^24^.

### Buffer layer in PSCs

**Fig. 2:**
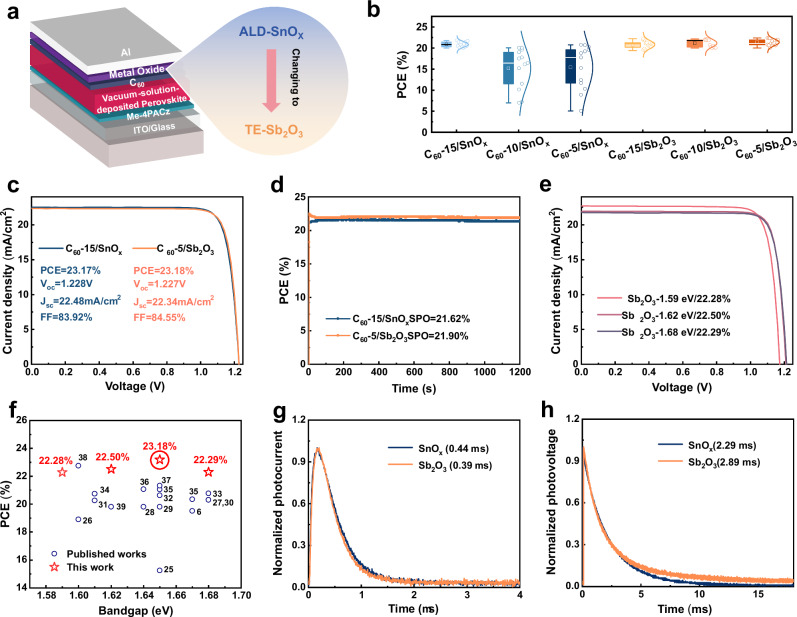
Photovoltaic performance of PSCs based on Sb_2_O_3_ and SnO_X_. **a** Schematic structure of PSCs. **b** Statistics of PCE of the PSCs with different C_60_ thicknesses based on SnO_X_ and Sb_2_O_3_, respectively. For the box plots, the central line denotes the median, and the square denotes the mean. The box bounds represent the 25th and 75th percentiles. The solid lines extending above and below the box represent the maximum and minimum values, respectively. The reverse-scan *J–V* curves (**c**) and SPO (**d**) of the 1.64 eV-bandgap champion PSCs based on SnO_X_ and Sb_2_O_3_ under AM1.5 G irradiation. e, The reverse-scan *J–V* curves of the 1.59, 1.62, 1.68 eV-bandgaps champion PSCs based on Sb_2_O_3_ under AM1.5 G irradiation. **f** Evolution of published PCE of mid/wide-bandgap PSCs prepared via vacuum-solution hybrid deposition^6,25–39^. TPC (**g**) and TPV (**h**) measurements of SnO_X_- and Sb_2_O_3_-based PSCs.

We applied Sb_2_O_3_ and SnO_X_ as buffer layer in p-i-n PSCs (Fig. 2a), respectively, utilizing triple-cation Cs_0.05_(MA_0.05_FA_0.95_)Pb(I_0.88_Br_0.12_)_3_ perovskite with a bandgap of 1.64 eV fabricated via vacuum-solution hybrid method. Notably, the configuration represents the first implementation of Sb_2_O_3_ as a charge-transport layer in photovoltaics. We modulated the C_60_ thickness via fixing the metal oxide thickness to 15 nm, as shown in Fig. 2b and Supplementary Fig. 19. The result of PV parameter distribution reveals a performance degradation with thinner C_60_ in the SnO_X_-based PSCs, marked by a sharp drop in fill factor (FF) at 5 nm. We attribute this primarily to the ligand exchange reaction between the TDMASn precursor and the perovskite (Supplementary Notes 4 and Supplementary Figs. 20–22). Besides, the 15 nm C_60_ layer functions as an effective barrier, preventing the irreversible interfacial reaction in the SnO_X_-based PSCs. In contrast, the Sb_2_O_3_-based PSCs present a broader process window for C_60_ thickness, owing to the non-destructive nature of TE-Sb_2_O_3_.

The Sb_2_O_3_ thickness is further adjusted to 15 nm by using 5 nm C_60_. We observed poor performance and repeatability for 5 nm Sb_2_O_3_ samples (Supplementary Fig. 23), which is ascribed to leakage current induced by non-denser 5 nm Sb_2_O_3_ (Supplementary Fig. 3). From reverse-scan *J*–*V* curve (Fig. 2c), the champion PSC, using 5 nm C_60_ and 15 nm Sb_2_O_3_, obtains a PCE of 23.18% with open-circuit voltage (*V*_OC_) of 1.227 V, *J*_SC_ of 22.34 mA/cm^−2^ and FF of 84.55%, which has similar PV parameter with 23.17% of champion PSC with 15 nm C_60_ and 15 nm SnO_X_. Amazingly, the PCE marks the highest efficiency reported for mid/wide-bandgap PSCs using vacuum-solution hybrid method^6,25–39^. The stabilized power output (SPO) of the champion PSCs with SnO_X_ and Sb_2_O_3_ also can be maintained at 22.74 and 23.02% after 600 s maximum power point (MPP) tracking, respectively (Fig. 2d). It illustrates that the Sb_2_O_3_ samples used by thin C_60_ layer still have excellent photovoltaic performance. We assess its universality via fabricated PSCs with 1.59, 1.62, and 1.68 eV-bandgaps perovskite absorbers. The 1.59, 1.62, 1.68 eV-bandgaps PSCs deliver notable performance with champion PCEs of 22.28, 22.50, and 22.29%, respectively (Fig. 2e and Supplementary Table 2), which are also among the highest PCE for the hybrid deposited PSCs^6,25–39^, as shown in Fig. 2f and Supplementary Table 3. All cells have achieved prominent FF, showing excellent generality in gently nature of Sb_2_O_3_. As shown in Supplementary Fig. 24, the integrated *J*_*SC*_ from the external quantum efficiency (EQE) spectra of PSCs with 1.59, 1.62, 1.64, and 1.68 eV-bandgaps matched well with the *J*_*SC*_ values from *J*–*V* curves (Fig. 2c, e).

To gain a deep insight in the electrical transport mechanism of Sb_2_O_3_, the transient photocurrent (TPC) and transient photovoltage (TPV) measurements were performed in Fig. 2g, h. The comparable photoelectron lifetimes of SnO_X_ and Sb_2_O_3_-based PSCs confirm the excellent efficiency of the defect-assisted carrier mechanism in Sb_2_O_3_. The similar photovoltage lifetimes of them indicates that the defect-assisted transport enables efficient charge extraction without introducing additional non-radiative recombination.

### Optical benefit in PSTs

**Fig. 3:**
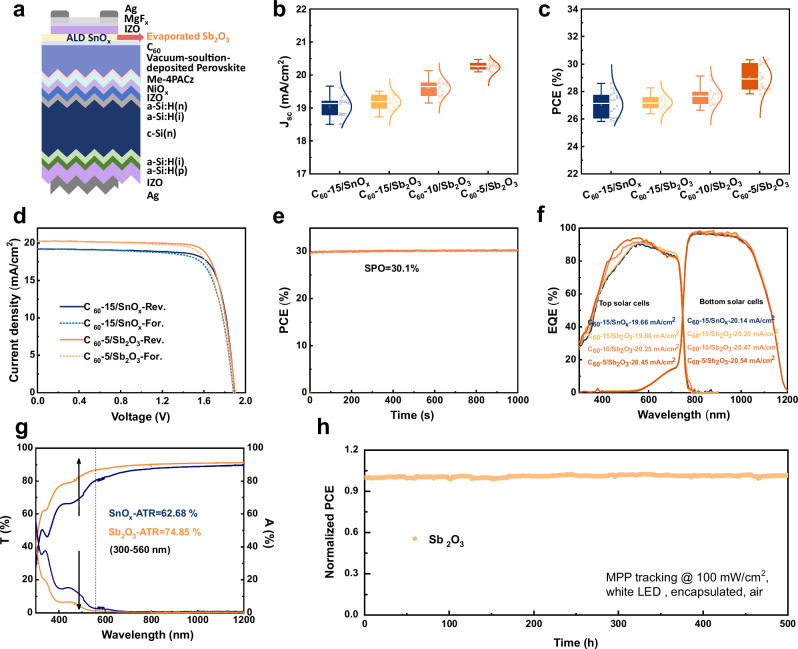
Photovoltaic performance and stability of PSTs based on Sb_2_O_3_ and SnO_X_. **a** Schematic of PST structure based on a double-side and sub-micron-pyramid-structured silicon heterojunction cell. Statistics of PCE (**b**) and *J*_SC_ (**c**) of the PSTs with Sb_2_O_3_ and SnO_x_. For the box plots, the central line denotes the median, and the square denotes the mean. The box bounds represent the 25th and 75th percentiles. The solid lines extending above and below the box represent the maximum and minimum values, respectively. *J–V* curves (**d**) and SPO (**e**) of the 1 cm^2^ champion PSTs. **f** EQE spectra of PSTs with Sb_2_O_3_ and SnO_X_. **g** Absorptance and transmittance spectra of C_60_/Sb_2_O_3_ and C_60_/SnO_X_ films. **h** Normalized *PCE* of encapsulated Sb_2_O_3_-based PST for MPP tracking under a white LED lamp illumination at 100 mW/cm^2^ in air.

To verify the adaptability of Sb_2_O_3_ in PSTs, we integrated the Sb_2_O_3_ film in 1 cm^2^ PSTs, which manufactured double-textured silicon heterojunction bottom cells and p-i-n structured perovskite top cells with 1.64 eV of bandgap (Fig. 3a and Supplementary Fig. 25).

Based on the electrical feasibility in single-junction devices (Fig. 2b), we fabricated PSTs by varying the thickness of C_60_ layers while fixing 15 nm Sb_2_O_3_ film. For direct comparison, control devices with 15 nm C_60_ and 15 nm SnO_X_ were prepared under identical conditions. The performance statistics of PSTs are summarized in Fig. 3b, c and Supplementary Fig. 26, wherein the gradually increased *J*_SC_ with thinning C_60_ for the tandem devices with Sb_2_O_3_, and more enhanced *J*_SC_ than the tandem device with SnO_X_ can be observed, facilitating to improving PCE. Among, the champion tandem device with Sb_2_O_3_ display a reverse-scan PCE of up to 30.28%, with a higher *J*_SC_ of 20.26 mA/cm^2^ than that of tandem device with SnO_X_ (19.22 mA/cm^2^), and almost the same *V*_OC_ of 1.894 V and FF of 78.67% (Fig. 3d and Supplementary Table 4). The SPO of 30.1% was also recorded after 2400 s MPP tracking (Fig. 3e).

Further, we conducted EQE and ultraviolet-visible (UV-vis) measurements to explain the reason of improving *J*_SC_ and color difference in aperture area (Fig. 3f, g and Supplementary Figs. 27, 28). The UV-vis shows reflection, transmittance and absorbance spectra of different thicknesses of C_60_. We detected that the average transmission (*T*_avg_) decreases from 81.17 to 63.87% in the wavelength range from 300 to 560 nm, with increasing C_60_ film thickness from 5 to 15 nm. As expected, the intensity of absorption increases with increasing film thickness. Therefore, the stronger EQE response of top sub-cell at the 300-560 nm wavelength is exhibited with thinning C_60_ film, resulting in a higher photocurrent of 20.45 mA/cm^2^ for C_60_ (5 nm)/Sb_2_O_3_ tandem device of top sub-cell and well-matched photocurrent of 20.54 mA/cm^2^ for bottom sub-cell (Fig. 3f). Next, UV-vis was employed to investigate the optical properties of C_60_/SnO_X_ and C_60_/Sb_2_O_3_ stacks (Fig. 3g and Supplementary Fig. 28). The C_60_ (5 nm)/Sb_2_O_3_ (15 nm) sample shows an *T*_avg_ of 74.85%, exceeding that of PVK/C_60_ (15 nm)/SnO_X_ (15 nm) sample (62.68%) in the wavelength range from 300 to 560 nm. Similarly, the intensity of absorption for C_60_ (5 nm)/Sb_2_O_3_ (15 nm) sample is lower than C_60_ (15 nm)/SnO_X_ (15 nm) sample, suggesting the achievement of lower parasitic light absorption by thinning C_60_.

To evaluate devices stability under operational conditions (Supplementary Notes 5 and Supplementary Fig. 29-39), we performed MPP tracking on encapsulated Sb_2_O_3_-based PST (Fig. 3h). After 500 h under white light emitting diode (LED) illumination (100 mW/cm^2^), The Sb_2_O_3_-based device exhibited negligible efficiency degradation. Furthermore, both encapsulated devices conducted the accelerated aging tests (LED illumination or 65 °C over 1000 h), retained over 90% of their initial PCE (Supplementary Figs. 37, 38).

### Application in encapsulated large-area PSTs

**Fig. 4:**
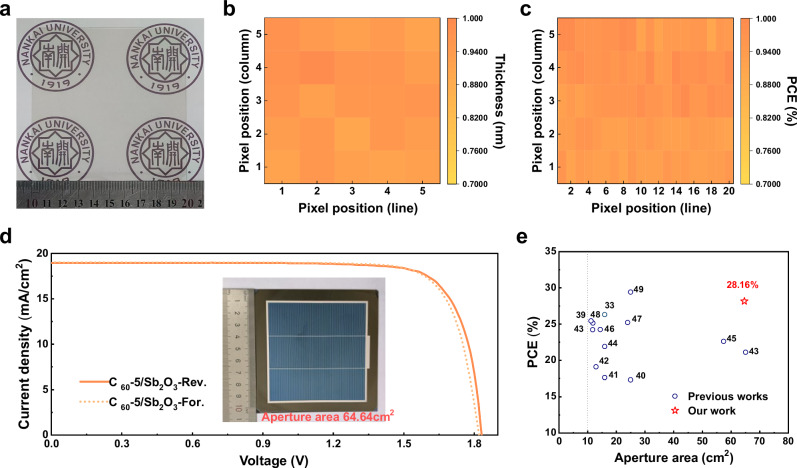
Photovoltaic performance of large-area tandem solar cells based on Sb_2_O_3_ and SnO_X_. **a** Photograph of Sb_2_O_3_ on a 10 × 10 cm^2^ glass substrate. **b** Statistical distribution of normalized thicknesses with 25 sub-samples on a 10 × 10 cm^2^ glass substrate. c, Statistical distribution of normalized PCE with 100 sub-cells based on Sb_2_O_3_ on a 10 × 10 cm^2^ substrate. **d** The *J–V* curves of the encapsulated large-area champion PST (64.64 cm^2^) with Sb_2_O_3_. e, Evolution of published PCE of PSTs ( > 10 cm²)^33,39–49^.

Scaling up aperture area in tandem devices represent a necessary trend for commercial development. To examine the homogeneity of Sb_2_O_3_, we deposited a 45 nm Sb_2_O_3_ film on a 10 × 10 cm^2^ glass substrate, which was subsequently divide it into 25 sub-samples, each measuring 2 × 2 cm^2^ (Fig. 4a). The results indicate nearly identical thickness across all samples, revealing superior uniformity of the large-area Sb_2_O_3_ film deposition in a 100 cm^2^ region (Fig. 4b and Supplementary Fig. 40**)**. Besides, we further selected five representative positions to verify the uniformity of C_60_ and the thinner Sb_2_O_3_ (15 nm) layers (Supplementary Notes 6 and Supplementary Figs. 41–43, Supplementary Table 5). The normalized PCE distribution of 100 sub-cells on a 10 × 10 cm^2^ substrate was also shown in Fig. 4c. Similarly, we fabricated Sb_2_O_3_ films as buffer layer in 100 sub-cells, adopting the device structure illustrated in Fig. 2a. The high similarity of the PCE for all sub-cells further proves the homogeneity of Sb_2_O_3_ film.

Furthermore, we used vacuum-solution hybrid method to fabricate 110.25 cm^2^ encapsulated PSTs with an aperture of 64.64 cm^2^ and achieved an enhanced PCE from 27.29 to 28.16% for Sb_2_O_3_-based PST, which is attributed to an improvement of *J*_SC_ from 18.61 to 18.96 mA/cm^2^ (Fig. 4d, Supplementary Fig. 44 and Supplementary Table 6). Crucially, The PCE of 28.16% is currently among the highest PCE for larger-area PSTs with the aperture area over 10 cm^2^ in published literature^33,39–49^ (Fig. 4e and Supplementary Table 7). Moreover, the encapsulated large-area PST was further certified by a third party (National Institute of Metrology) showed a reverse-scan PCE of 27.70% (Supplementary Fig. 45). Furthermore, a SPO of 27.66% was confirmed following 1800 s of stabilization at the MPP (Supplementary Fig. 46). In addition, the process demonstrates excellent reproducibility across multiple independent fabrication, indicating promising scalability (Supplementary Notes 7, Supplementary Fig. 47 and Supplementary Table 8). Notably, the development of Sb_2_O_3_ achieves dual breakthroughs in performance and cost, which reduces fabrication cost to ~70% of conventional ALD-SnO_X_, demonstrating significant commercial potential (Supplementary Notes 8 and Supplementary Table 9).

## Disscussion

In this work, we firstly developed an amorphous-nanocrystalline Sb_2_O_3_ film via thermal evaporation to serve as an alternative buffer layer to ALD-deposited SnO_X_, where the embedded nanocrystals form effective electron transport pathways. The Sb_2_O_3_ film exhibits exceptional optical transmission, high sputtering tolerance, and excellent compatibility with ultra-thin C_60_ (5 nm), enabling non-destructive perovskite integration. Using a vacuum-solution hybrid processing approach, Sb_2_O_3_-based PSCs with tunable bandgaps (1.59, 1.62, 1.64, and 1.68 eV) achieved PCEs of 22.28, 22.50, 23.18, and 22.29%, respectively. Notably, the 23.18% PCE currently represents the highest reported value for mid/wide-bandgap PSCs fabricated using the vacuum-solution hybrid method. Furthermore, Sb_2_O_3_-based PSTs have achieved a champion PCE of 30.28% for 1.0 cm^2^ devices, surpassing the 28.69% efficiency demonstrated by SnO_X_-based PSTs. The 64.64 cm² encapsulated PSTs with Sb_2_O_3_ buffer layer demonstrate outstanding performance, achieving a champion PCE of 28.16% (with a certified PCE of 27.70%). This represents one of the highest reported efficiencies for larger-area PSTs with an aperture area exceeding 10 cm² in published literature. Enabled by Sb_2_O_3_ films, the optical parasitic loss from C_60_ in PSTs is further reduced, paving the way for efficiencies beyond 35% in the future.

## Methods

### Materials

Me-4PACz (>99%) and C_60_ were purchased from Lumtec. Ethanol (99.5% anhydrous) and isopropyl alcohol (IPA) (99.5% anhydrous) were purchased from Alfa Aesar. Lead iodide (PbI_2_, >99.99%) and antimony trioxide (Sb_2_O_3_, >99.9%) were purchased from Sigma-Aldrich. Formamidine bromide (FABr), formamidine iodide (FAI), methylamine hydrochloride (MACl), Cesium chloride (CsCl), 4-fluorobenzylamine hydroiodide (F-PMAI) and 1,3-propanediammonium iodide (PDADI) were purchased from Xi’an Polymer Light Technology (China).

### Fabrication of silicon bottom cells

We employed a fabrication process for the silicon heterojunction (SHJ) bottom cell that closely followed established methods reported in the literature^5^. The procedure is as follows: an n-type < 100 > CZ Si wafer with a resistivity of 1–5 Ω·cm was used. An initial etching step was conducted to remove saw damage and polish the wafer. Subsequently, both the front and rear surfaces of the wafer were mildly textured using a relatively low-concentration alkaline solution to ensure uniform small-pyramid structures with a size of 300–500 nm on both sides. After standard cleaning and a hydrogen fluoride dip, hydrogenated intrinsic amorphous silicon layers ( ~ 5 nm thick) were deposited on both sides. Then, an n-type hydrogenated nanocrystalline silicon oxide layer ( ~ 15 nm) and a p-type hydrogenated nanocrystalline silicon (nc-Si:H) layer ( ~ 20 nm) were deposited on the front and rear, respectively. To complete the bottom cell, an 80-nm-thick In_2_O_3_-based transparent conductive oxide followed by silver was deposited on the rear, while a 10-nm-thick TCO film was deposited on the front. The electrode area was 1.1 × 1.1 cm^2^, and the silicon cells were laser-cut to 2.03 × 2.03 cm^2^ substrates for tandem fabrication.

### Fabrication of single-junction PSCs

The single-junction devices were fabricated with the structure: ITO/Me-4PACz/Perovskite/PDADI/C_60_/SnO_X_ or Sb_2_O_3_/Al. Glass substrates with ITO coating were cleaned with three solvents in the sequence of deionized water, acetone, and ethanol. The Me-4PACz solution in ethanol solution was spin-coated at 3000 rpm for 30 s, following an annealing treatment at 100 °C for 10 min in N_2_ glovebox. Then, the Me-4PACz film was clean up via spin-coating 100 µL ethanol at 5000 rpm for 30 s. CsCl was evaporated in a Lesker mini Spectros system chamber (working pressure < 5 × 10^−6 ^mbar, evaporation rates of 0.4 Å/s). Next, PbI_2_ was co-evaporated with CsCl to form inorganic precursor in same chamber (working pressure < 5 × 10^−6 ^mbar, evaporation rates of 2 Å/s for PbI_2_ and of 0.1 Å/s for CsCl). The substrate temperature was kept at 30 °C. Subsequently, a solution of FABr and FAI with different molar ratio of 1:5.2, 4.6, 1.5, 0.8 (bandgaps of 1.59, 1.62, 1.64, 1.68 eV) dissolved in ethanol and containing 10 mol% of MACl and 5 mol% of F-PMAI, was spin-coated at 5000 rpm for 30 s in N_2_ glovebox, following by the annealing step at 150 °C for 20 min in ambient air with a 30-35% humidity. PDADI dissolved in IPA (1 mg mL^−1^) was spin-coated onto perovskite layer at 5000 rpm for 30 s, following an annealing treatment at 100 °C for 5 min in N_2_ glovebox. Afterward, different thicknesses of C_60_ (5, 10, 15 nm) and of Sb_2_O_3_ (5, 15, 25 nm) were sequentially evaporated in a homemade evaporation system to fabricate Sb_2_O_3_-based PSCs (working pressure < 5 × 10^−6^ mbar, substrate holder at 30 °C). For the PSCs with SnO_x_, the Sb_2_O_3_ is replaced by 15 nm SnO_x_ through ALD at 80 °C using TDMASn and hydrogen peroxide as precursors. Finally, the metal grids were fabricated by depositing a 80 nm thick layer of Al (Angstrom Engineering) through thermal evaporation, using a shadow mask. Besides, the active area was 0.0755 cm^2^ for single-junction devices.

### Fabrication of PSTs

Twenty nm nickel oxide (NiO_X_) layer was sputtered using a nickle target at room temperature in pure Ar at 80 W to cap the ITO recombination layer on the top of SHJ bottom cells. Then, Me-4PACz, perovskite, PDADI, C_60_ and Sb_2_O_3_ or are deposited on NiO_X_ substrate using same process as the single-junction PSCs, where the perovskite with 1.64 eV bandgap is used in small-area tandem devices (1 cm^2^), the perovskite with 1.59 eV bandgap is used in large-area tandem devices (64.64 cm^2^). Subsequently, 50 nm of IZO transparent electrode was sputtered in a Lesker sputtering system using a 6-in. target (90% In_2_O_3_ and 10% ZnO) with a radio frequency power of 38 W (sheet resistance of 40 Ω/sq). Afterward, 200 nm of Ag was evaporated through a shadow mask to form the metal electrode for small-area tandem devices (1 cm^2^), and the screen-printing metal grids instead of the evaporated Ag electrode for large-area tandem devices (64.64 cm^2^), following the curing of silver paste was annealing at 85 °C for 20 min.

### Device characterization

*J*–*V* curves of single-junction PSCs and tandem solar cells were measured by a Keithley 2400 Source meter under Enli Solar Simulator (AM 1.5 G, 100 mW cm^−2^), calibrated by a standard silicon reference cell. The devices were measured with a scan rate of 20 mV/s, the voltage step of 0.02 V, and delay time of 40 ms. During the J–V test, shadow masks with area of 0.0755 cm^2^ for single-junction and of 1 or 64.64 cm^2^ for tandem solar cells were used, respectively. EQE of single-junction PSCs were measured using an Enli Tec (Taiwan) system. For tandem solar cells, EQE spectra were measured by a QEX10 PV Measurement system in the wavelength from 300 to 1200 nm with a scanning rate of 10 nm. SPO was measured in N_2_ glovebox at room temperature with multichannel system (Enli. Tech.) under AM 1.5 G illumination by a xenon-lamp. Device stability was evaluated through aging tests under LED illumination using a high-power LED light source system (CEL-LED100HA-96, AuLight) and full-spectrum AM 1.5 G illumination provided by a xenon-lamp solar simulator (FG-MN001-ACA-AM1.5G-100, FengGuan), each calibrated to 100 mW/cm^2^. Encapsulated tandem devices were subjected to continuous light soaking at ~40 °C under both illumination conditions, with periodic *J*–*V* measurements to track the evolution of MPP efficiency over time.

### Other characterization

XPS and UPS were performed by Thermo Scientific ESCALAB 250Xi spectrometer. UV-vis spectra was carried out through a Varian Cary 500 spectrophotometer. SEM images was characterized by FEINanoSEM650 with an acceleration voltage of 1 kV. AFM, kelvin probe force microscopy (KPFM) and c-AFM tested by Bruker Dimension Icon with Pt/Ir coated conductive probes. Grazing-incidence X-ray diffraction (GIXRD) patterns of films were obtained by a multifunctional diffractometer (Rigaku, ATX-XRD) with Cu Kα radiation (*λ* = 1.5405 Å). The cross-sectional TEM samples were prepared using a focused ion beam (FIB) lift-out technique on a ZEISS Crossbeam 540 FIB-SEM system. TEM imaging and selected area electron diffraction analysis were performed using an FEI Tecnai F20 field-emission transmission electron microscope operating at an acceleration voltage of 200 kV. Dark *J*–*V* curve measurement was conducted in dark in dark by Keithley 2400 Source meter at a scan rate of 20 mV/s with a delay time of 20 ms, where the bias scan ranged from −2 to 2 V. TPC and TPV were obtained with a digital oscilloscope (DOS-X 304 A).

### Reporting summary

Further information on research design is available in the Nature Portfolio Reporting Summary linked to this article.

## Supplementary information

Supplementary Information

Reporting Summary

Transparent Peer Review file

## Source data

Source data

## Data Availability

Source data are provided with this paper. All other data of this work are available from the corresponding authors on request. Source data are provided with this paper.
