# Supplementary material for: Antimony oxide buffer layer for single- and double-junction perovskite-based solar cells
Source: Nat Commun. 2026 Mar 25;17:4394. doi: 10.1038/s41467-026-70848-8 (PMC13181109; doi:10.1038/s41467-026-70848-8)
Supplement: Supplementary file 1 — Supplementary Information [file 41467_2026_70848_MOESM1_ESM.pdf]

Supplementary Information

Antimony Oxide Buffer Layer for Single- and Double-Junction Perovskite-Based Solar Cells

Biao Shi‡[1,2,3,4,5], Zetong Sunli‡[1,2,3,4,5], Pengfei Liu[1,2,3,4,5], Wei Han[1,2,3,4,5], Rui Kong[1,2,3,4,5], Cong Sun[1,2,3,4,5], Ying Liu[1,2,3,4,5], Yuan Luo[1,2,3,4,5], XianZhao Wang[1,2,3,4,5], Zhi Zhang[1,2,3,4,5], Dekun Zhang[1,2,3,4,5], Xiaona Du[1,2,3,4,5], Fu Zhang[6], Miao Yang[6], Yongcai He*[6], Bo He[6], Xixiang Xu[6], Rui Xia[7], Xueling Zhang[7], Yifeng Chen[7], Jifan Gao*[7] , Fuzong Xu[8], Ying Zhao[1,2,3,4,5], Stefaan De Wolf*[8], and Xiaodan Zhang*[1,2,3,4,5]

** heyongcai@longi.com, Jifan.gao@trinasolar.com, stefaan.dewolf@kaust.edu.sa, xdzhang@nankai.edu.cn*

[1] Institute of Photoelectronic Thin Film Devices and Technology, Renewable Energy Conversion and Storage Center, State Key Laboratory of Photovoltaic Materials and Cells, Nankai University, Tianjin 300350, P. R. China

[2] Tianjin Key Laboratory of Efficient Utilization of Solar Energy, Tianjin 300350, P. R. China

[3] Haihe Laboratory of Sustainable Chemical Transformations, Tianjin 300192, P. R. China

[4] Engineering Research Center of Thin Film Photoelectronic Technology of Ministry of Education, Tianjin 300350, P. R. China

[5] Collaborative Innovation Center of Chemical Science and Engineering (Tianjin), Tianjin 300072, P. R. China

[6] LONGi Central R&D Institute, LONGi Green Energy Technology Co., Ltd., Xi'an,710018, China

[7] State Key Laboratory of PV Science and Technology, Trina Solar, Changzhou 210031, China

[8] King Abdullah University of Science and Technology (KAUST), Physical Sciences and Engineering Division (PSE), Material Science and Engineering Program (MSE), Thuwal, Kingdom of Saudi Arabia.

B.S. and Z.S. contributed equally to this work.

Supplementary Notes

Supplementary Note 1: Defect-Assisted Electron Transport Mechanism in Sb_2O_3

The antimony oxide (Sb_2O_3) films demonstrate a nanocrystal-mediated electron transport mechanism, where the embedded nanocrystals act as conductive pathways for efficient vertical charge transport across the Sb_2O_3 film. Considering the requirements for device integration, the wide bandgap of Sb_2O_3 (4.25 eV) tends to create energy barriers that hinder interfacial electron extraction (**Supplementary Figure 6**). Ultraviolet-visible (UV-vis) spectra of 100 nm Sb_2O_3 film showed substantial sub-bandgap absorption, confirming abundant in-gap states[1,2] (**Supplementary Figure 7**). We propose that these gap states could potentially mediate efficient electron transport across the interface[1,3].

The hypothesis was systematically examined through a combination of correlated experiments and theoretical calculations. A controlled annealing experiment was conducted (**Supplementary Figure 7**): the 100 nm Sb_2O_3 film was annealed at 350 °C under N_2 atmosphere, which resulted in negligible change in its sub-bandgap absorption. In contrast, the Sb_2O_3 film annealed in air dramatically suppressed the sub-bandgap absorption, implicating oxygen-related defects (e.g. interstitial Sb, I_{Sb}) as the source. Also, X-ray photoelectron spectrometry (XPS) confirmed the presence of Sb^0 atoms, which serve as precursors for the formation of I_{Sb} (**Supplementary Figure 8**). A systematic first-principles calculations of the cubic-Sb_2O_3 band structure was performed with the aim of identifying the dominant defect species by introducing various types of defects (**Supplementary Figures 9-10**). Crucially, it can be observed that the I_{Sb} defects introduce specific in-gap states: two acceptor levels (E_{A1} at 1.06 eV, E_{A2} at 1.86 eV) and a donor level (E_D at 2.90 eV). Systematic correlation of measured absorption difference spectra ($\Delta\alpha$, the difference between the two absorption spectra) with theoretical calculations provides compelling evidence for defect-mediated charge transport. The $\Delta\alpha$ reveals inflection points at 2.2, 2.74, and 3.27 eV, corresponding to calculated E_{A2}→CBM (E_2=2.18 eV), VBM→E_D (E_3=2.90 eV), and E_{A1}→CBM (E_1=2.98 eV) transitions in whole VBM→CBM transition, respectively (**Supplementary Figures 7 and 11**). Besides, the monotonically decreasing $\Delta\alpha$ between 2.2 and 3.27 eV demonstrates the formation of quasi-continuous defect state around these I_{Sb} levels. This band architecture enables efficient electron injection across the 1.2 eV fullerene (C_{60})/Sb_2O_3 interface barrier via defect-assisted percolation (**Supplementary Figures 12-13**). In addition, the transition from discrete theoretical levels to continuous experimental bands is rationally explained by collective environmental effects in real material systems, where high defect density and local fluctuations induce significant state broadening and hybridization.

Supplementary Note 2: First-Principles Calculations

All first-principles calculations based on density functional theory (DFT) were carried out using the Vienna Ab initio Simulation Package (VASP)[4]. The electron-ion interactions were described by the projector augmented-wave (PAW) method[5]. Considering the van der Waals nature of the Sb_2O_3 crystal, the r^2SCAN+rVV10 vdW-DF method was employed to describe the exchange-correlation effects during the structural optimization[6]. The obtained lattice constant of the cubic phase is 11.09 Å, which is almost identical to the reported experimental data of 11.10 Å[7]. For the calculation of band structure and related properties, the meta-generalized gradient approximation (meta-GGA) level with the MBJ functional was employed[8]. The calculated direct (4.20 eV) and indirect (4.04 eV) band gaps show excellent agreement with the experimental optical band gap of 4.25 eV. An energy cutoff of 500 eV was applied for the plane-wave basis set expansion. A Γ center k-point mesh of 5×5×5 was used for structural optimization calculations. The convergence criteria were set to 1×10^{-5} eV for total energy and to 0.05 eV/Å for atomic forces in optimizations, respectively.

Supplementary Note 3: Contact Property and Interfacial Stress of C_{60}/Sb_2O_3

Given the critical influence of residual stress on the properties and stability of thin films, quantifying this stress is essential for evaluating their performance and reliability. The C_{60} (30 nm)/Sb_2O_3 sample fabricated without high-temperature processing (substrate holder at 30 °C) was characterized by optical microscopy. The absence of interlayer cracks, warping, or delamination confirms the low intrinsic stress of the Sb_2O_3 film (**Supplementary Figure 16**). Moreover, the microstructure of the C_{60}/Sb_2O_3 sample was examined by grazing-incidence X-ray diffraction (GIXRD) in **Supplementary Figure 17**. By tuning the incident angle θ_{inc} from 0.3° to 3°, the probe depth was varied from the surface to the deeper regions of the film[9,10], which was confirmed by the emergence of a broad diffraction signal from the amorphous glass/C_{60} substrate at θ_{inc}=3°. Notably, the X-ray diffraction (XRD) peaks showed no shift with depth, thereby enabling direct assessment of the film stress state. The constant peak positions confirm negligible stress gradient within the Sb_2O_3 film and poor residual at the Sb_2O_3/C_{60} interfaces, which is purely a physical contact mediated by van der Waals forces.

Supplementary Note 4: ALD-SnO_X Induced Perovskite Degradation and Non-destructive Evaporated-Sb_2O_3

Atomic layer-deposited tin oxide (ALD-SnO_X) is typically fabricated using tetrakis(dimethylamino)tin(IV) (TDMASn) and hydrogen peroxide as precursors[11,12]. Previous studies reported that the TDMASn precursor undergoes a severe ligand exchange reaction with the perovskite, driven by the displacement of its dimethylamine groups by formamidinium ions[11]. As evidenced by the *N 1s* XPS spectrum (**Supplementary Figure 20**), a distinct peak at ~398.5 eV appears alongside the perovskite signal at ~400.6 eV, indicating the formation of reduced nitrogen species through Sn–N bonding[12,13]. No obvious changes were detected in the *I 3d* and *Pb 4f* orbitals. In addition, the appearance of PbI_2 diffraction peaks in the XRD pattern[14] (**Supplementary Figure 21**) indicates the decomposition of the perovskite lattice due to interfacial reactions. No significant morphological changes were observed in scanning electron microscopy (SEM) images (**Supplementary Figure 22**), suggesting the degradation occurs at a microscopic/chemical level. In contrast, thermal evaporated Sb_2O_3 is a purely physical process involving sublimation and condensation, without complex precursors or post-deposition annealing[7]. This inherent simplicity prevents any chemical interaction with the perovskite layer, as confirmed by XPS and XRD results (**Supplementary Figures 21-22**).

Combined on the single-junction devices with various C_{60} thicknesses (**Figure 2b and Supplementary Figure 19**), the SnO_X-based devices with thinner C_{60} layers exhibit inferior performance, attributed to insufficient protection against the ligand exchange reaction. However, the thicker C_{60} effectively suppresses interfacial degradation, thereby enhancing device performance and confirming its additional role as a protective barrier. In contrast, the evaporated Sb_2O_3-based devices can be compatible with thinner C_{60}, offering greater flexibility in optical management of tandem cells.

Supplementary Note 5: Stability of Sb_2O_3-Based Films and Devices

To evaluate the stability of Sb_2O_3-based devices, we systematically examined the intrinsic Sb_2O_3 and Sb_2O_3-based stack layers, full devices based on Sb_2O_3. Considering the application of encapsulated devices in real operation, the stability tests were conducted under air-free conditions, focusing on light stability (under a 100 mW/cm^2 white LED) and thermal stability (at 65 °C), respectively. The Sb_2O_3 film showed excellent intrinsic photothermal stability, which exhibits no significant change of the peak information before and after aging in XPS spectra (**Supplementary Figure 29**). The transmission electron microscope (TEM) and XRD results also show no obvious crystal structure change over a 40-day aging period (**Supplementary Figures 30-31**).

For Sb_2O_3-based stack layers, the C_{60}/Sb_2O_3 interface exhibits excellent stress stability, as confirmed by GIXRD measurements, showing no peak positions shifting across the film depth after aging, attribute to simple physical interaction via van der Waals (**Supplementary Figure 31**). Also, the stability of PVK/C_{60}/SnO_X or Sb_2O_3/IZO stacks was investigated in **Supplementary Figures 32-34**, revealing no noticeable decomposition or morphological degradation for aged perovskite layer. XRD patterns further confirmed the intact perovskite structure (**Supplementary Figure 35**), indicating no observable adverse effects of Sb_2O_3 on the perovskite layer.

The encapsulated Sb_2O_3-based perovskite/silicon tandem solar cells (PSTs) were subjected to 500 hours of MPP tracking under a white LED lamp illumination at 100 mW/cm^2 (**Supplementary Figure 36**). The Sb_2O_3-device maintained their initial power conversion efficiency (PCE) without substantial loss. In addition, we conducted comparative studies (encapsulated SnO_X vs. Sb_2O_3-based PSTs), and continuously monitored the photothermal stability for more than 1,000 hours under light (white LED lamp illumination at 100 mW/cm^2) and 65 °C thermal aging tests, respectively (**Supplementary Figures 37-38**). Both encapsulated devices retaining over 90% of their initial PCE. To further evaluate the stability under realistic illumination conditions, we performed continuous aging tests under AM 1.5G illumination (xenon lamp solar simulator, 100 mW/cm^2). As shown in **Supplementary Figure 39**, after 450 hours, the encapsulated Sb_2O_3-based device retained ~65% of its initial PCE, while the SnO_X-based device retained ~80%.

Supplementary Note 6: Uniformity of Large-Area C_{60} and Sb_2O_3 Films

The high uniformity of the functional layers are critical for achieving high efficiency in large-area perovskite solar cells. Cross-sectional TEM image of the PVK/C_{60}/Sb_2O_3/IZO stack is presented in **Figure 1g**, intuitively demonstrating the continuous coverage provided by the 5 nm C_{60} and 15 nm Sb_2O_3 layers. We have also verified the excellent uniformity of 40 nm Sb_2O_3 on a 100 cm^2 substrate (**Supplementary Figure 40**). The homogeneity of the 15 nm Sb_2O_3 layer was assessed by depositing it on a 100 cm^2 large-area substrate. Next, atomic force microscopy (AFM) measurements were performed at five representative positions on the Sb_2O_3 film, revealing a consistent step height of approximately 15 nm (**Supplementary Figure 41**). Moreover, the similar surface potential (~700 mV) of these five sub-samples further confirm the superior uniformity of the 15 nm Sb_2O_3 film (**Supplementary Figure 42**). Similarly, the homogeneity of the large-area C_{60} layer was further evaluated through UV-vis measurements at five representative positions on a 100 cm^2 substrate coated with a 5 nm film (**Supplementary Figure 43** and **Supplementary Table 5**). The similar transmittance reveal the superior coverage uniformity of 5 nm C_{60} film at the large-area scale.

Supplementary Note 7: Reproducibility and Scalability of Large-Area PSTs

The reproducibility and scalability of the large-area Sb_2O_3-based PSTs (64.64 cm^2) were evaluated through the performance of devices fabricated in multiple independent batches. Four large-area tandem devices were fabricated separately across four batches under identical conditions. As shown in **Supplementary Figure 47** and **Supplementary Table 8**, these devices exhibit an average PCE of 27.82% with a remarkably low relative standard deviation (RSD) of 1.52%. The RSDs for other key parameters (open-circuit voltage V_{OC}, short-circuit current density J_{SC}, and fill factor FF) are all below 2%. The minimal performance variation across batches unequivocally confirms the excellent reproducibility and robustness of the fabrication process. This outcome provides critical data supporting its reliable scalability from laboratory-scale to larger areas.

Supplementary Note 8: Economic Advantage of Sb_2O_3 Buffer Layers in Large-Area Tandem Solar Cells

SnO_X is the prevailing buffer layer material in state-of-the-art PSTs [15-17]. Replacing SnO_X with Sb_2O_3 results in higher short-circuit current density and power conversion efficiency in tandem solar cells, and the parameters in the main text demonstrate the technical feasibility of using Sb_2O_3 as a buffer layer. Taking the highest-efficiency cell in the text as an example, the conventional combination is 15 nm C_{60}/15 nm SnO_X, while the innovative combination in this work is 5 nm C_{60}/15 nm Sb_2O_3.

Currently, the techno-economic analysis of tandem cells uses a bottom-up model to predict the levelized cost of electricity (LCOE)[18-20]. The cost of each process step includes materials, equipment, labor, utilities, maintenance, building and facilities costs. However, this analysis method is highly influenced by factors such as region, policies, and production line capacity, making the model complex and subject to continuous updates as production line designs evolve[20]. Given that the tandem cell in this study only modified the C_{60} and buffer layer, we calculated the material cost, equipment depreciation cost, electricity cost, and sum of them for the different combination of C_{60} and buffer layer on a 100 cm^2 silicon substrate in our laboratory (**Supplementary Table 9**). The total cost of 15 nm C_{60}/15 nm SnO_X is about 3.4 times that of 5 nm C_{60}/15 nm Sb_2O_3 for each experiment, demonstrating the economic feasibility of using Sb_2O_3 as a buffer layer. ([a]Note that material utilization factor is closely related to factors such as equipment type, geometry, and deposition process. In this statistics, thin films were deposited according to steps in the experimental procedure, and the material loss for each experiment was determined by weighing the source material before and after deposition using a balance. The average material utilization factor was obtained through film quality divided by material loss from three different film thickness experiments. [b]Note that the unit prices of materials and equipment investments are based on the costs of the products and equipment used in our laboratory. [c]Note that the processing duration of evaporated film and ALD-deposited SnO_X is the minimum total time of evacuating the chamber, heating, stabilizing and depositing film.)

Supplementary Figures

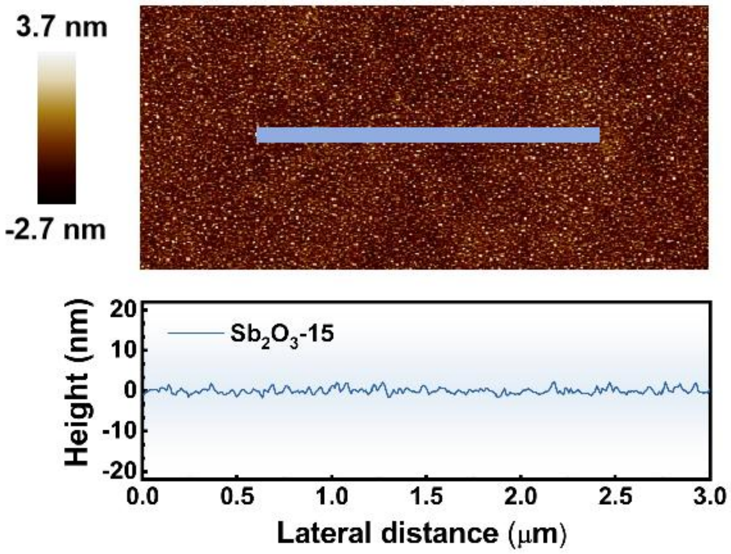

Supplementary Figure 1 AFM image of Sb_2O_3 and corresponding height profile.

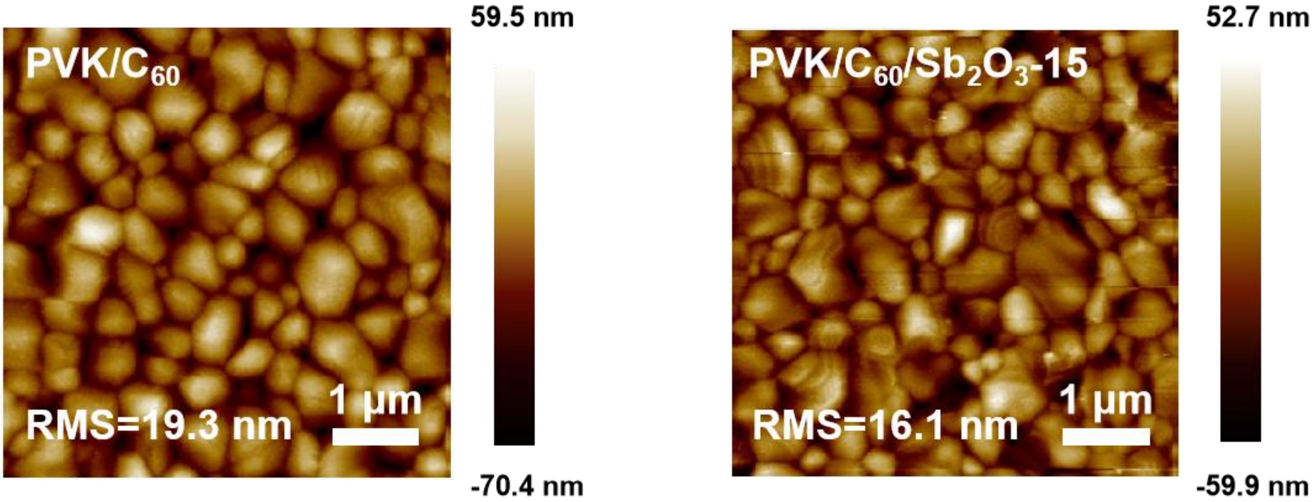

Supplementary Figure 2 AFM images of PVK/C_{60}/without and with Sb_2O_3.

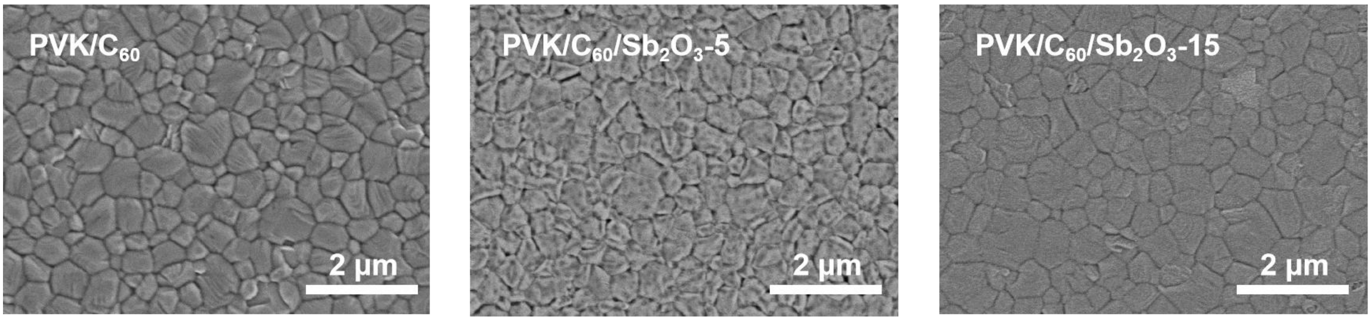

Supplementary Figure 3 SEM images of PVK/C_{60}/without and with Sb_2O_3.

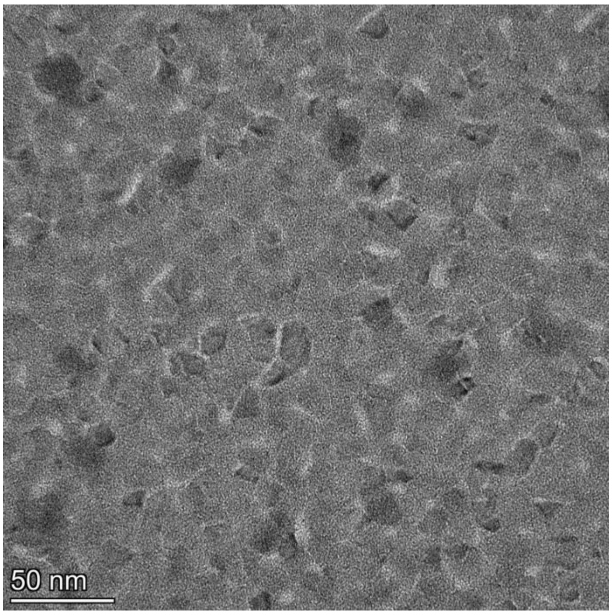

Supplementary Figure 4 TEM image of Sb_2O_3 film.

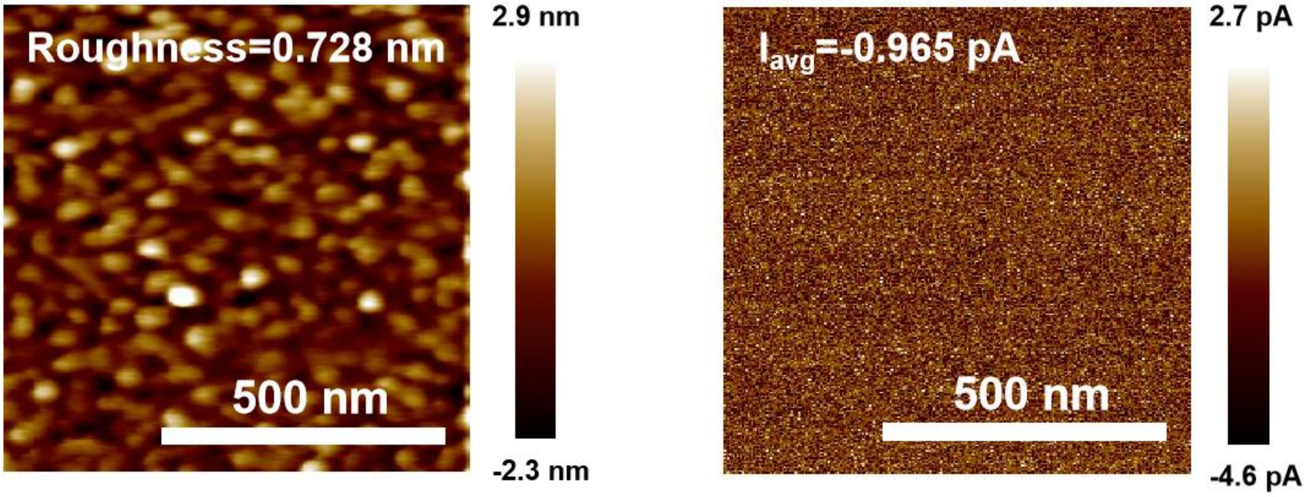

Supplementary Figure 5 AFM and corresponding lateral conductive atomic force microscopy (c-AFM) images of Sb_2O_3 film under a bias voltage of 3 V.

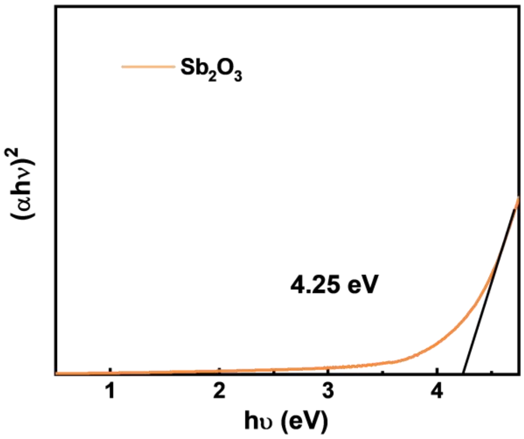

Supplementary Figure 6 Tauc-plot of Sb_2O_3 film.

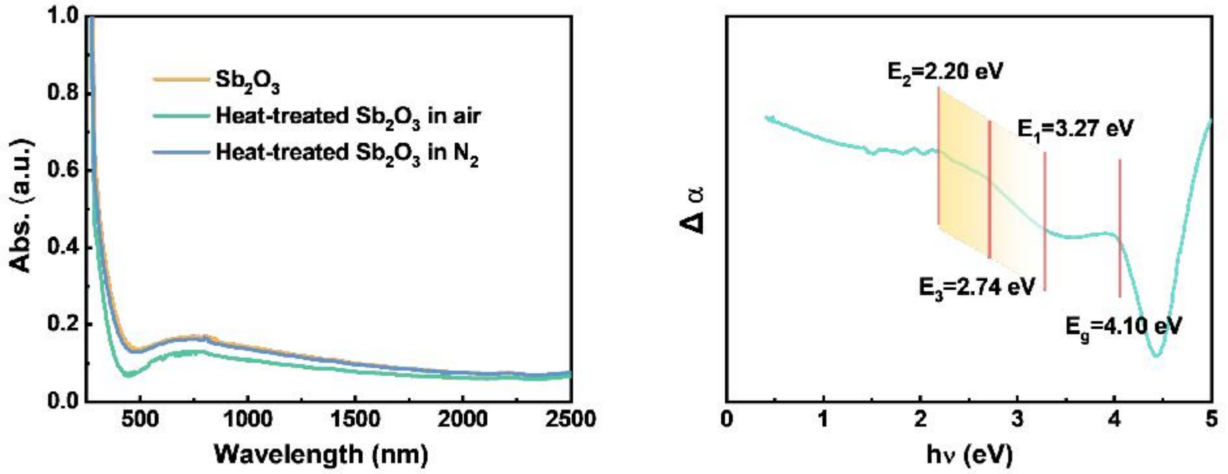

Supplementary Figure 7 UV-vis absorption spectra of 100 nm Sb_2O_3 films under different processing conditions: (i) as-deposited, (ii) after annealing in N_2 at 350 °C, and (iii) after annealing in air at 350 °C; The difference in absorption spectrum (air-annealed minus as-deposited samples).

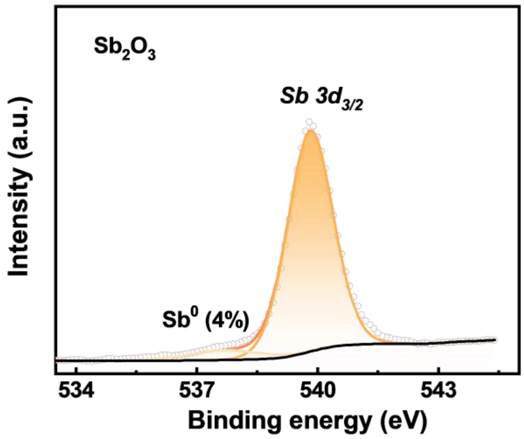

Supplementary Figure 8 XPS spectrum of *Sb 3d*$_{3/2}$ of Sb_2O_3 film.

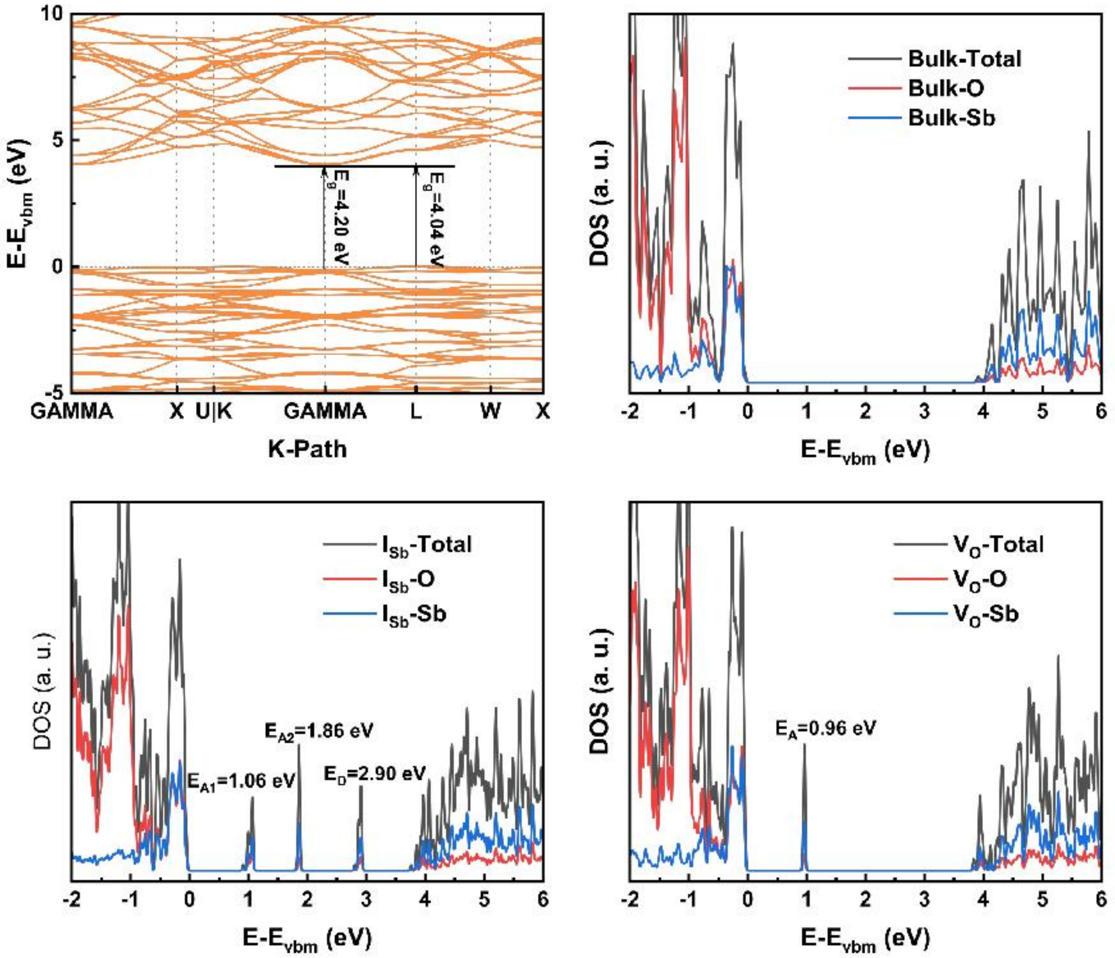

Supplementary Figure 9 Electronic band structures of perfect cubic Sb_2O_3 and defective systems with various point defects calculated by DFT (e.g., Sb interstitials, I_{Sb} or oxygen vacancies, V_O).

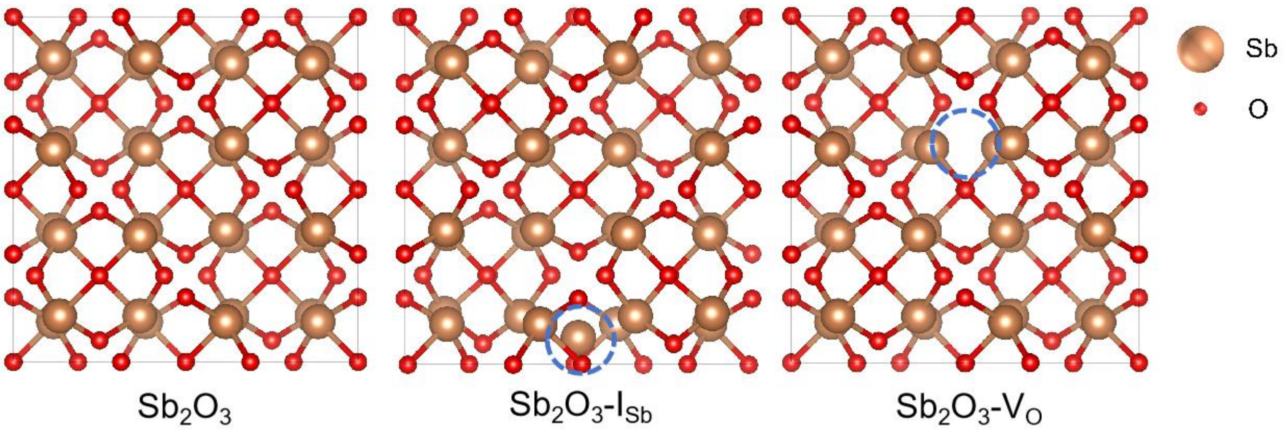

Supplementary Figure 10 Top views of the Sb_2O_3 crystal and defect structures (e.g. I_{Sb} and V_O).

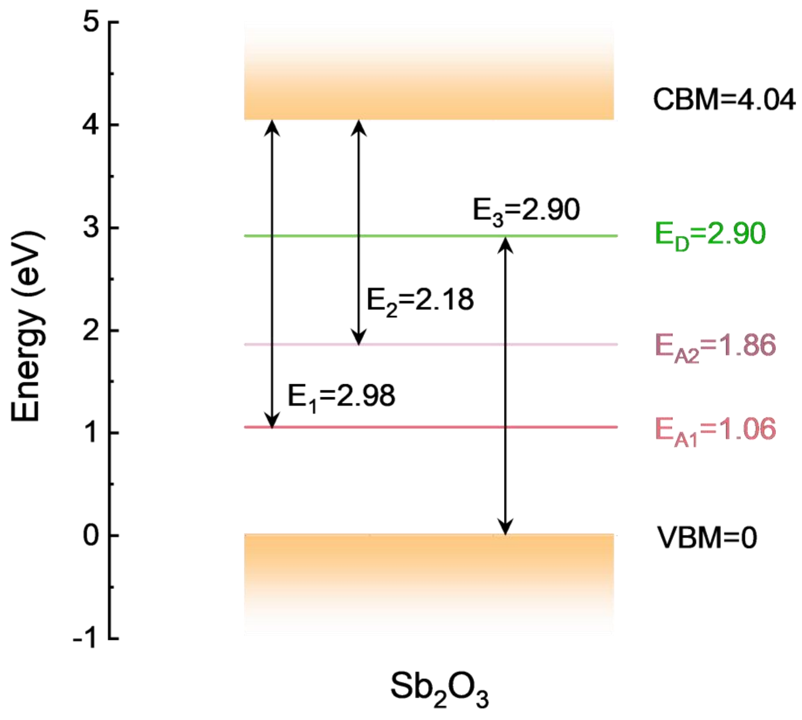

Supplementary Figure 11 Energy level structure and optical transitions induced by I_{Sb} defects. Schematic diagram of the Sb_2O_3 energy levels upon introduction of I_{Sb}, obtained from DFT calculations. The optical transition energies E_1 and E_2 from the acceptor levels E_{A1} and E_{A2} to the CBM, and E_3 from the VBM to the donor level E_D are indicated.

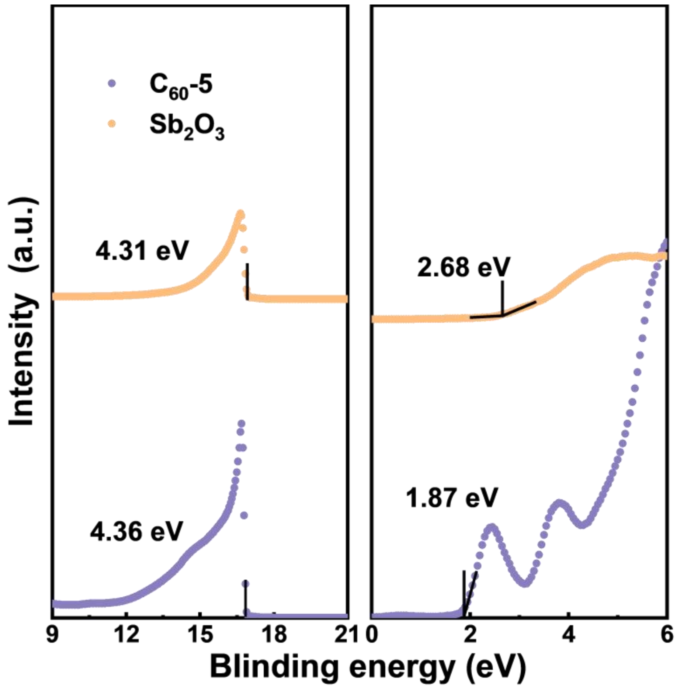

Supplementary Figure 12 Ultraviolet photoelectron spectroscopy (UPS) spectra of C_{60} and Sb_2O_3 films.

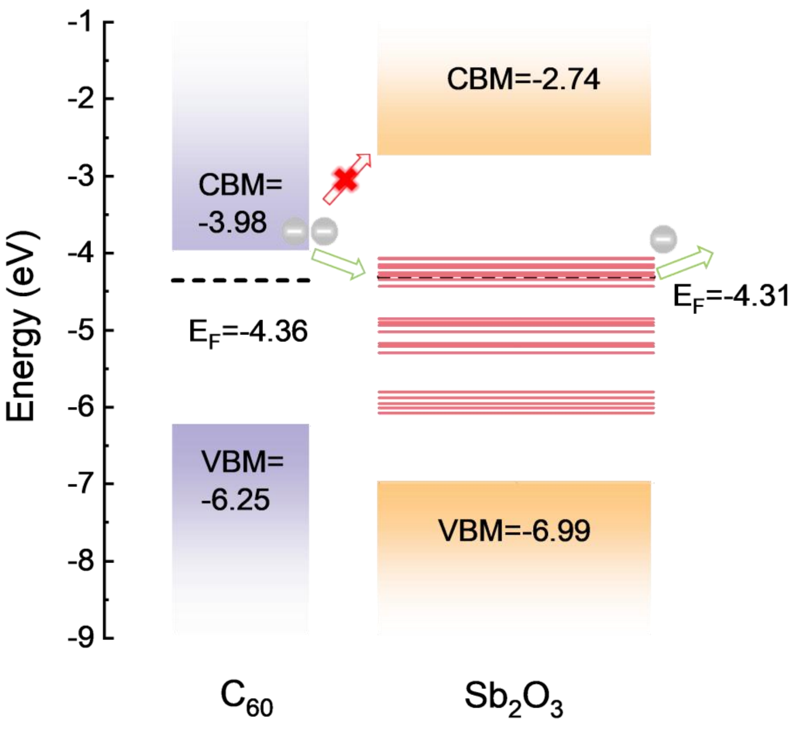

Supplementary Figure 13 Schematic energy level alignment and electron transport mechanism at the C_{60}/Sb_2O_3 interface. The energy level diagram of C_{60} and Sb_2O_3, based on UPS results, illustrates that electrons can be efficiently transported from the LUMO of C_{60} to the Sb_2O_3 layer via a quasi-continuous defect band induced by I_{Sb}, despite the large conduction band offset (~1.2 eV).

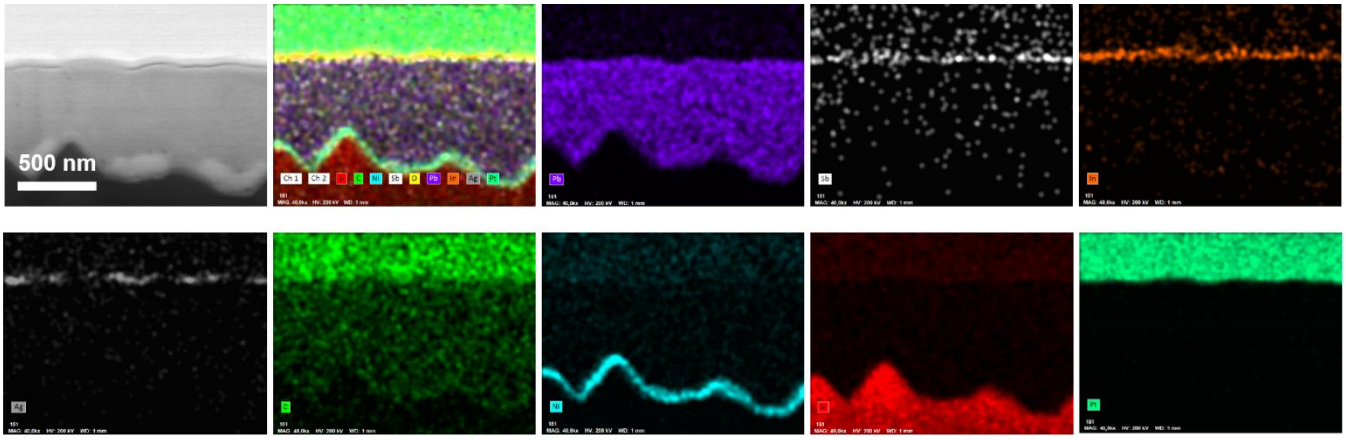

Supplementary Figure 14 Cross-sectional TEM image and energy dispersive spectrometer (EDS) mapping images of Si/NiO_X/Me-4PACz/PVK/C_{60}/Sb_2O_3/IZO stack films.

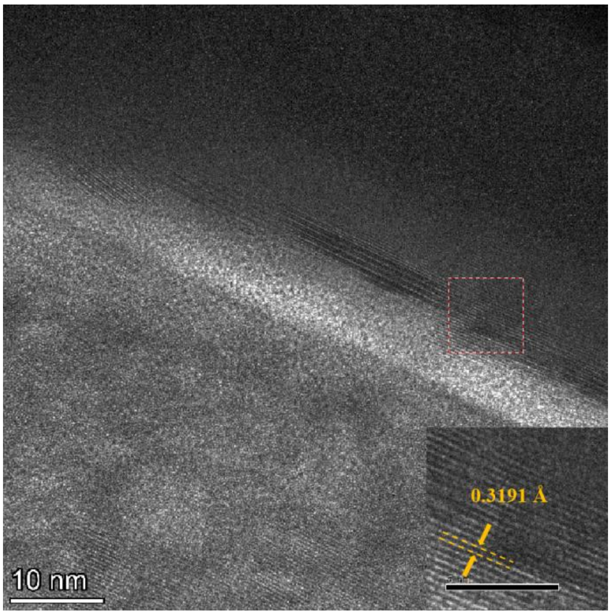

Supplementary Figure 15 Cross-sectional TEM image of C_{60}/Sb_2O_3/IZO stack film.

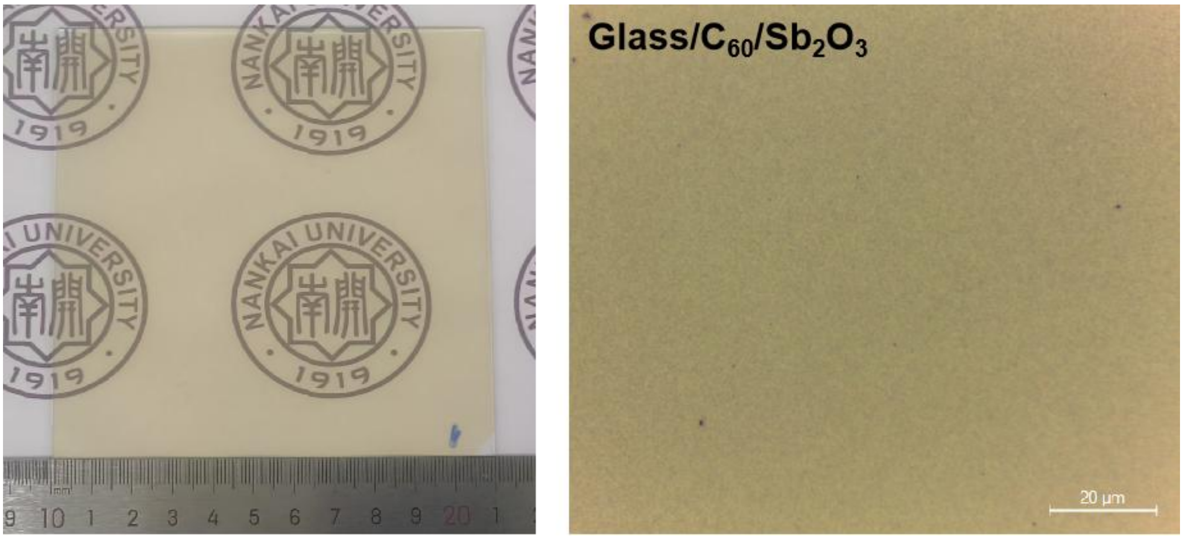

Supplementary Figure 16 Photograph and optical microscopy of the Glass/C_{60}-25/Sb_2O_3 samples.

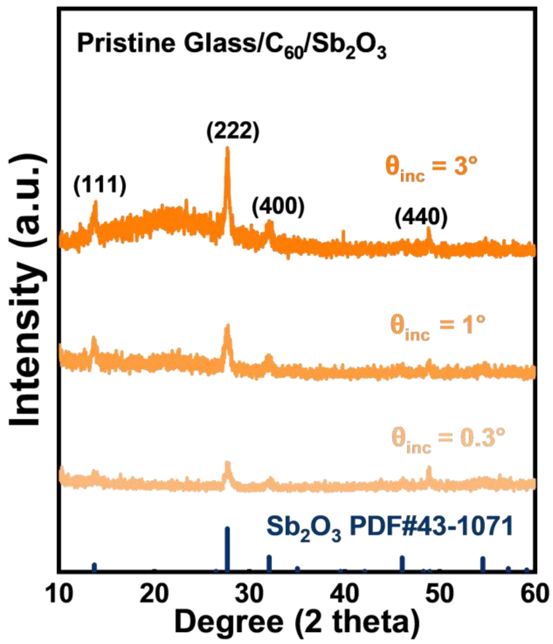

Supplementary Figure 17 GIXRD patterns of Glass/C_{60}-25/Sb_2O_3 samples at different incidence angles (θ_{inc}=0.3, 1 and 3°).

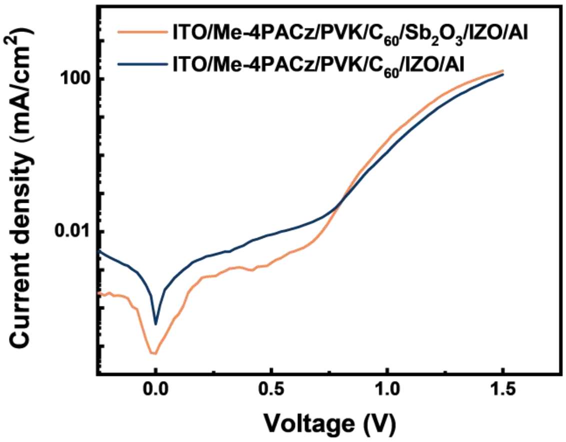

Supplementary Figure 18 Dark *J-V* curve of the structure with ITO/Me-4PACz/PVK/C_{60}/with or without Sb_2O_3/IZO/Al.

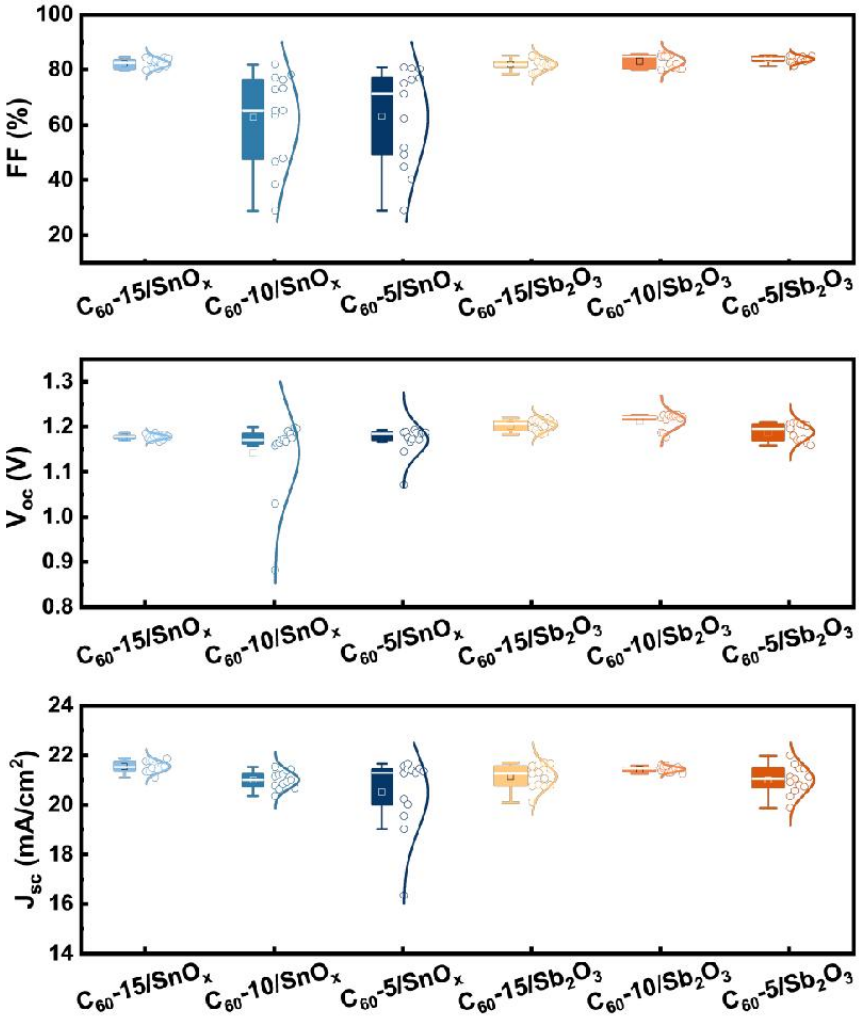

Supplementary Figure 19 Statistics of V_{OC}, J_{SC} and FF of the PSCs with different C_{60} thicknesses based on SnO_X and Sb_2O_3. For the box plots, the central line denotes the median, and the square denotes the mean. The box bounds represent the 25th and 75th percentiles. The solid lines extending above and below the box represent the maximum and minimum values, respectively.

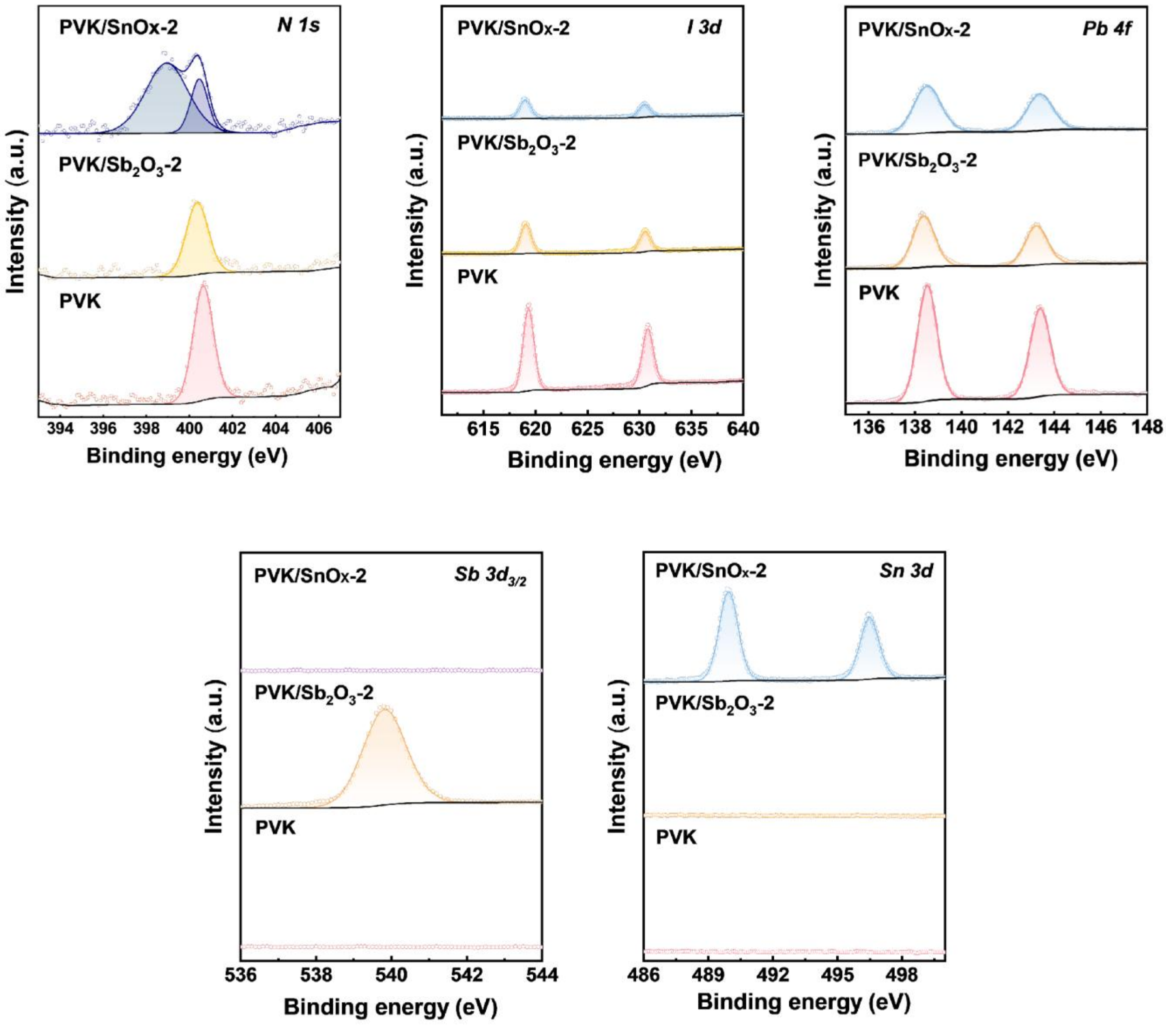

Supplementary Figure 20 XPS spectra of core levels (*N 1s*, *I 3d*, *Pb 4f*, *Sb* $3d_{3/2}$, *Sn 3d*) for the pristine PVK and PVK/SnO_X or Sb_2O_3.

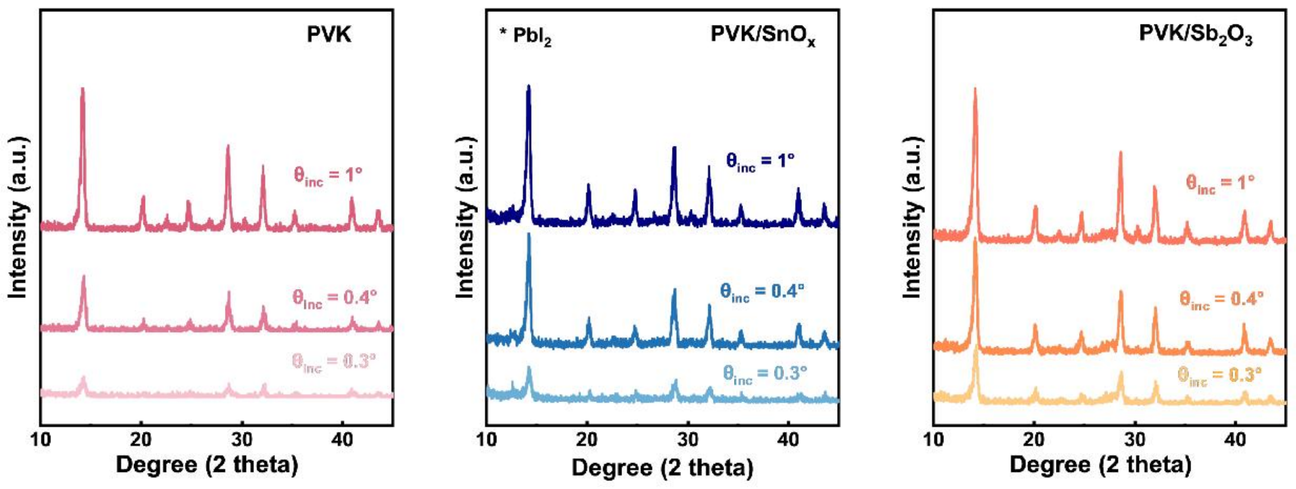

Supplementary Figure 21 GIXRD patterns of pristine PVK and PVK/SnO_X or Sb_2O_3. at different incidence angles (θ_{inc}=0.3, 0.4 and 1°).

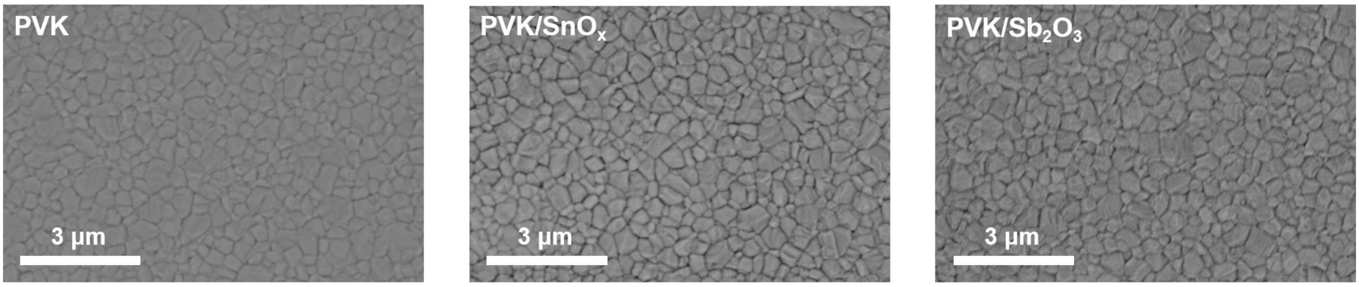

Supplementary Figure 22 SEM images of pristine PVK and PVK/SnO_X or Sb_2O_3.

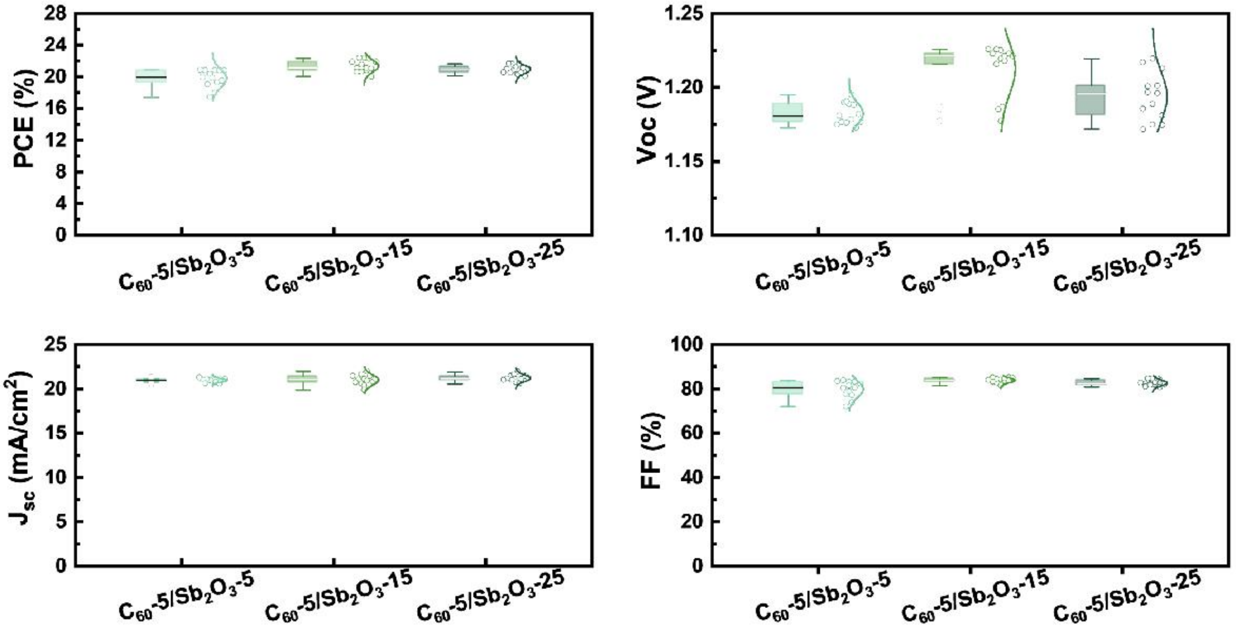

Supplementary Figure 23 Statistics of PCE, V_{OC}, J_{SC} and FF of the PSCs with different Sb_2O_3 thicknesses. For the box plots, the central line denotes the median, and the square denotes the mean. The box bounds represent the 25th and 75th percentiles. The solid lines extending above and below the box represent the maximum and minimum values, respectively.

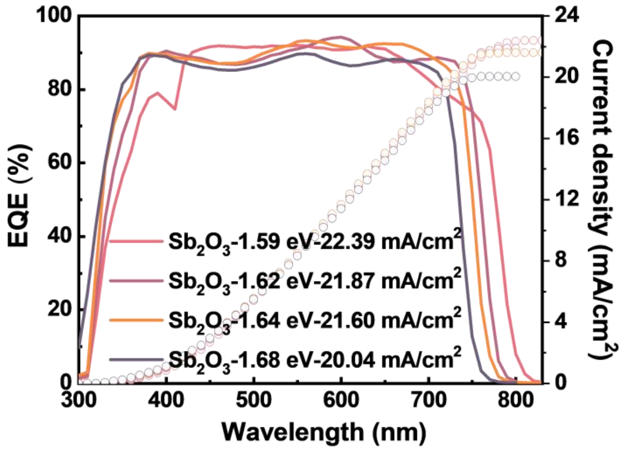

Supplementary Figure 24 External quantum efficiency (EQE) spectra and integrated current density of 1.59, 1.62, 1.64 and 1.68 eV-bandgaps PSCs.

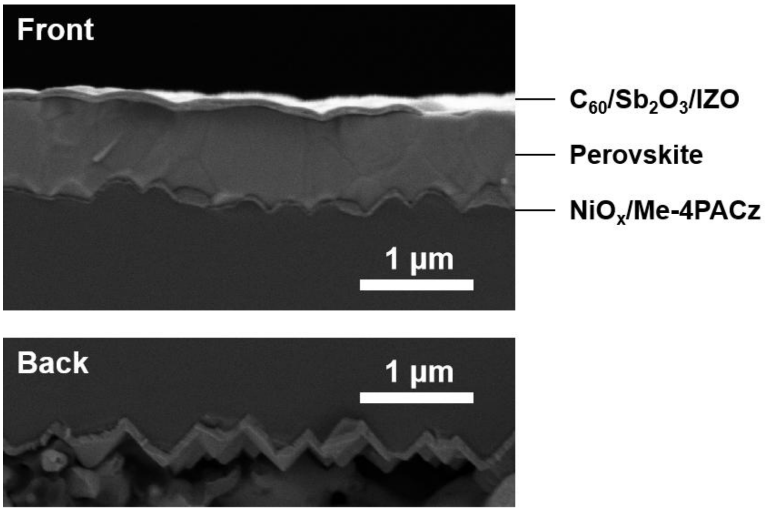

Supplementary Figure 25 Cross-sectional SEM images of PST structure.

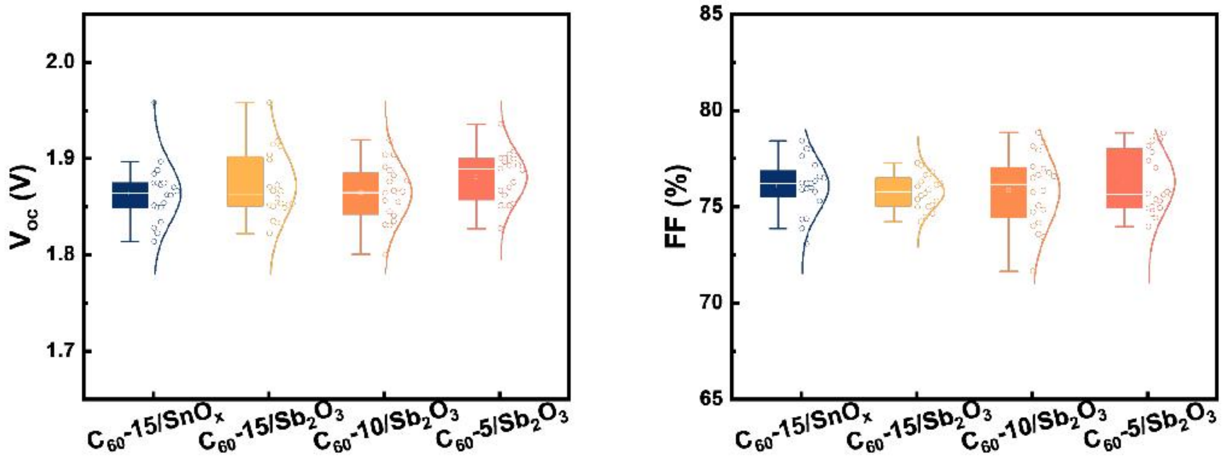

Supplementary Figure 26 Statistics of V_{OC}, FF of the PSTs with SnO_X and Sb_2O_3. For the box plots, the central line denotes the median, and the square denotes the mean. The box bounds represent the 25th and 75th percentiles. The solid lines extending above and below the box represent the maximum and minimum values, respectively.

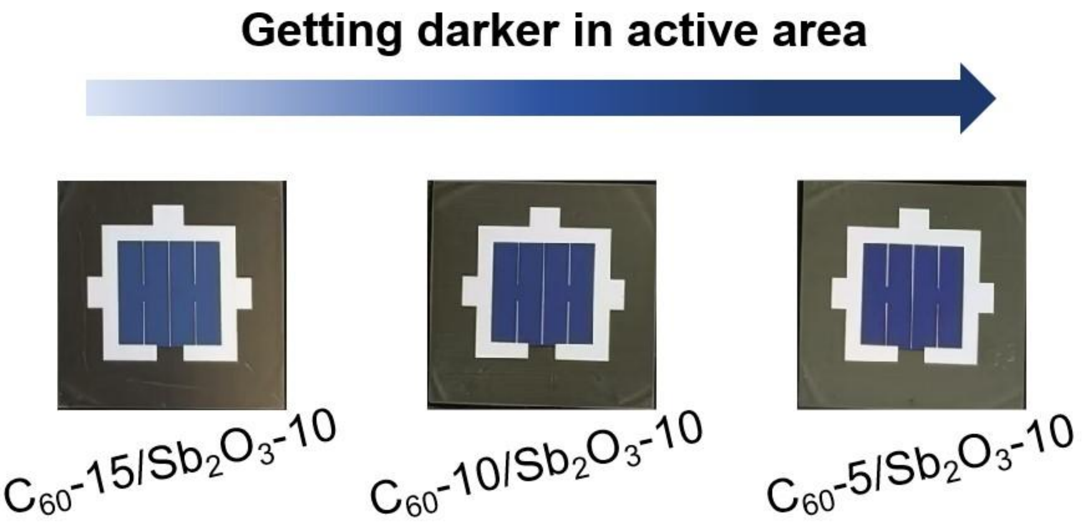

Supplementary Figure 27 Photographs of tandem solar cells with different C_{60} thicknesses based on Sb_2O_3.

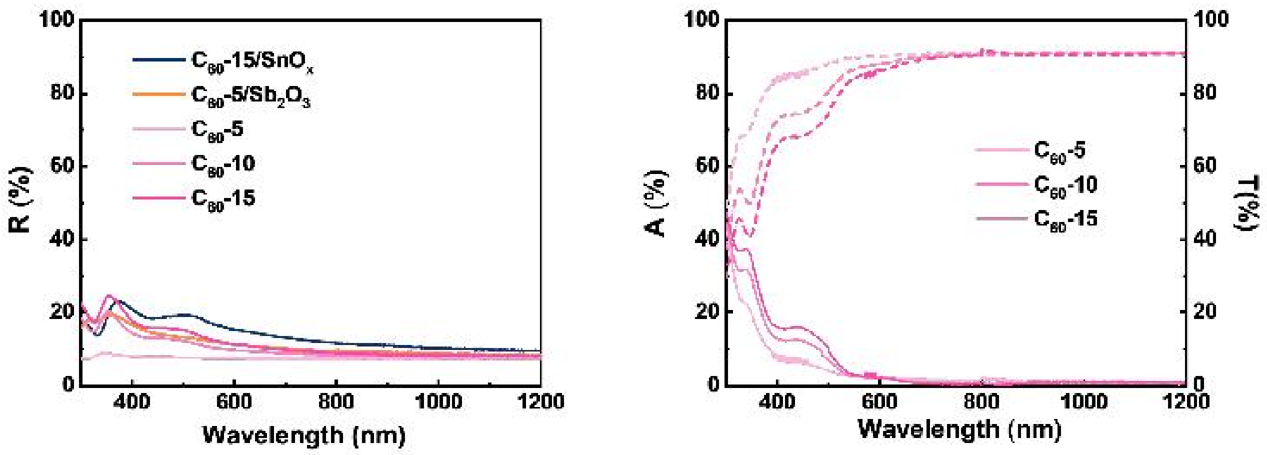

Supplementary Figure 28 Reflection, absorptance and transmittance spectra of C_{60}/SnO_X or Sb_2O_3 and pure C_{60} films with different thickness.

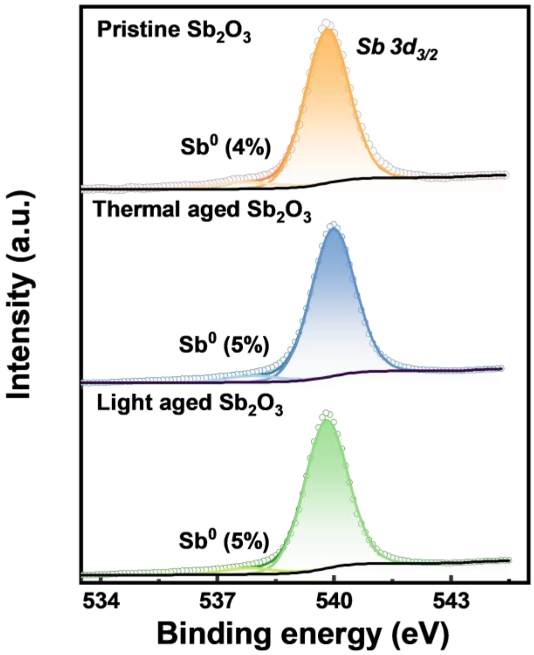

Supplementary Figure 29 XPS spectra of Sb_2O_3 films under different aging conditions. (i) Pristine film, and films aged after 40 days in N_2 atmosphere under (ii) 65 °C or (iii) a white LED source (100 mW/cm^2).

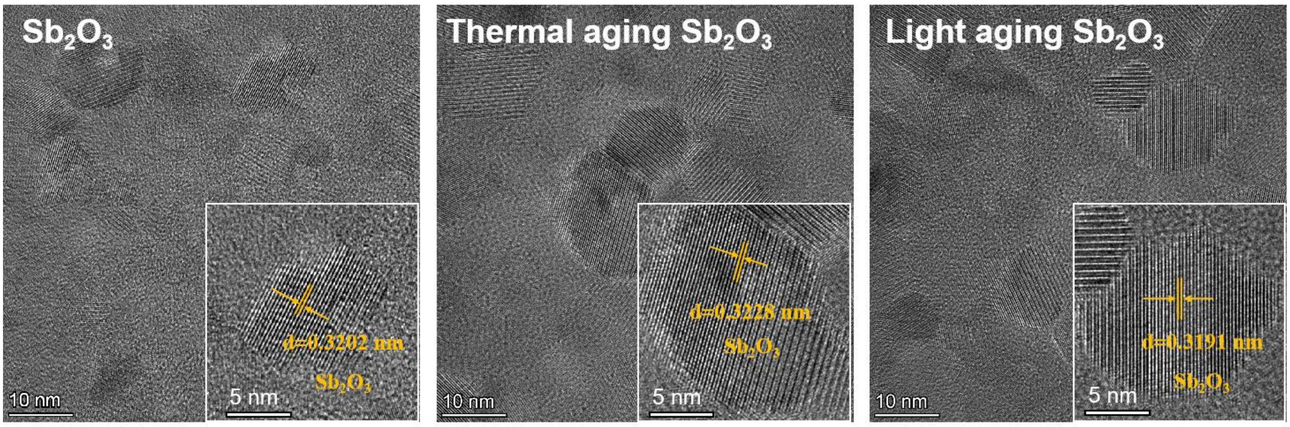

Supplementary Figure 30 Transmission electron microscope (TEM) images of Sb_2O_3 films under different aging conditions. (i) Pristine film, and films aged after 40 days in N_2 atmosphere under (ii) 65 °C or (iii) a white LED source (100 mW/cm^2).

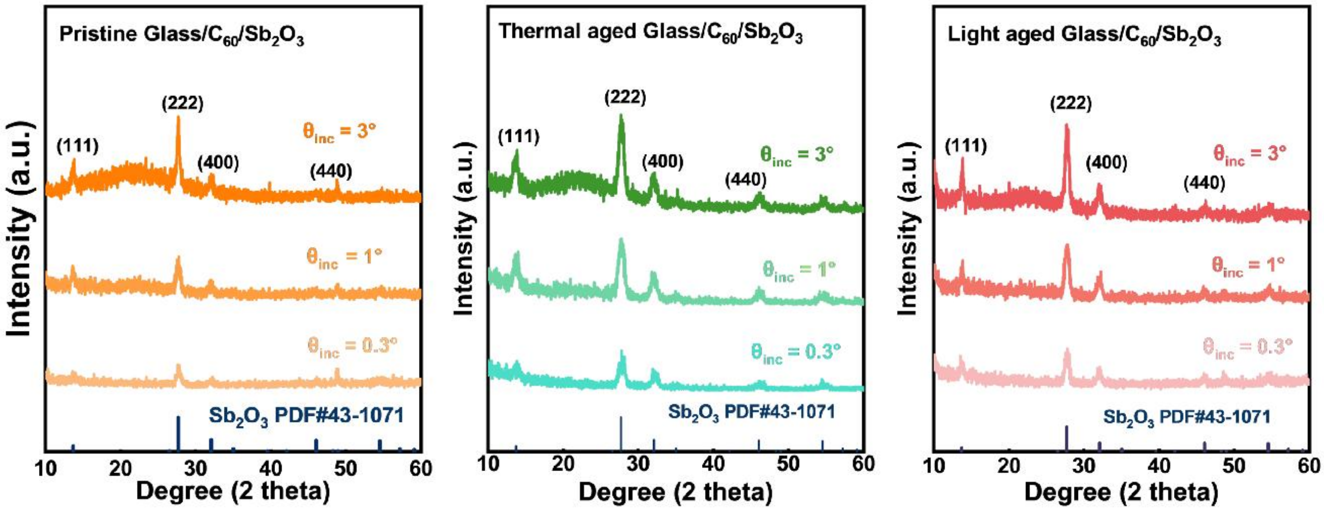

Supplementary Figure 31 GIXRD patterns (θ_{inc}=0.3, 1 and 3°) of Glass/C_{60}/Sb_2O_3 films under different aging conditions. (i) Pristine film, and films aged after 40 days in N_2 atmosphere under (ii) 65 °C or (iii) a white LED source (100 mW/cm^2).

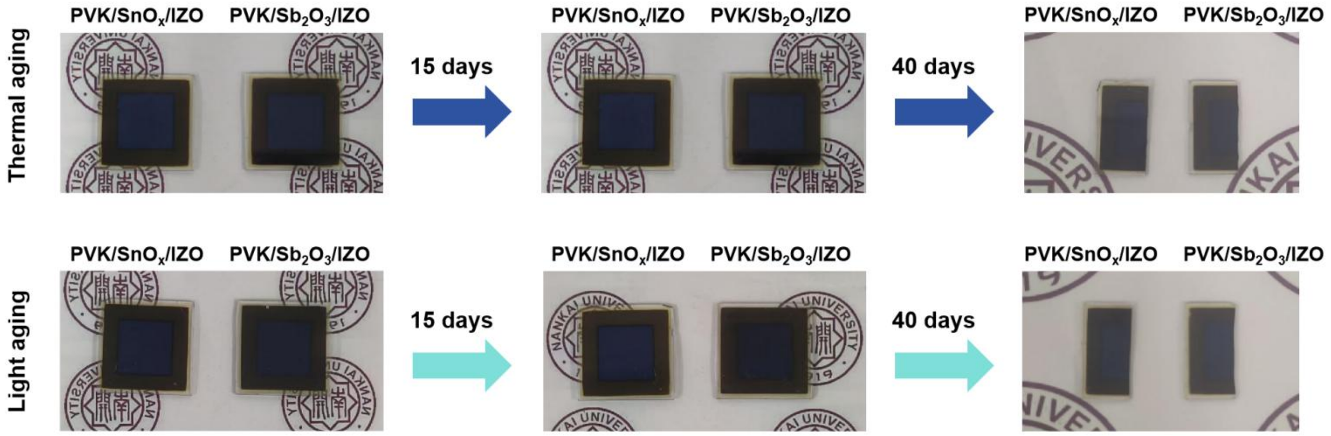

Supplementary Figure 32 Photographs of Glass/Me-4PACz/PVK/C_{60}/SnO_X- or Sb_2O_3/patterned IZO stacks under different aging conditions. (i) Pristine film, and films aged after different days in N_2 atmosphere under (ii) 65 °C or (iii) a white LED source (100 mW/cm^2). (Note: These sample areas were reduced after 40 days due to sample sectioning for characterization.)

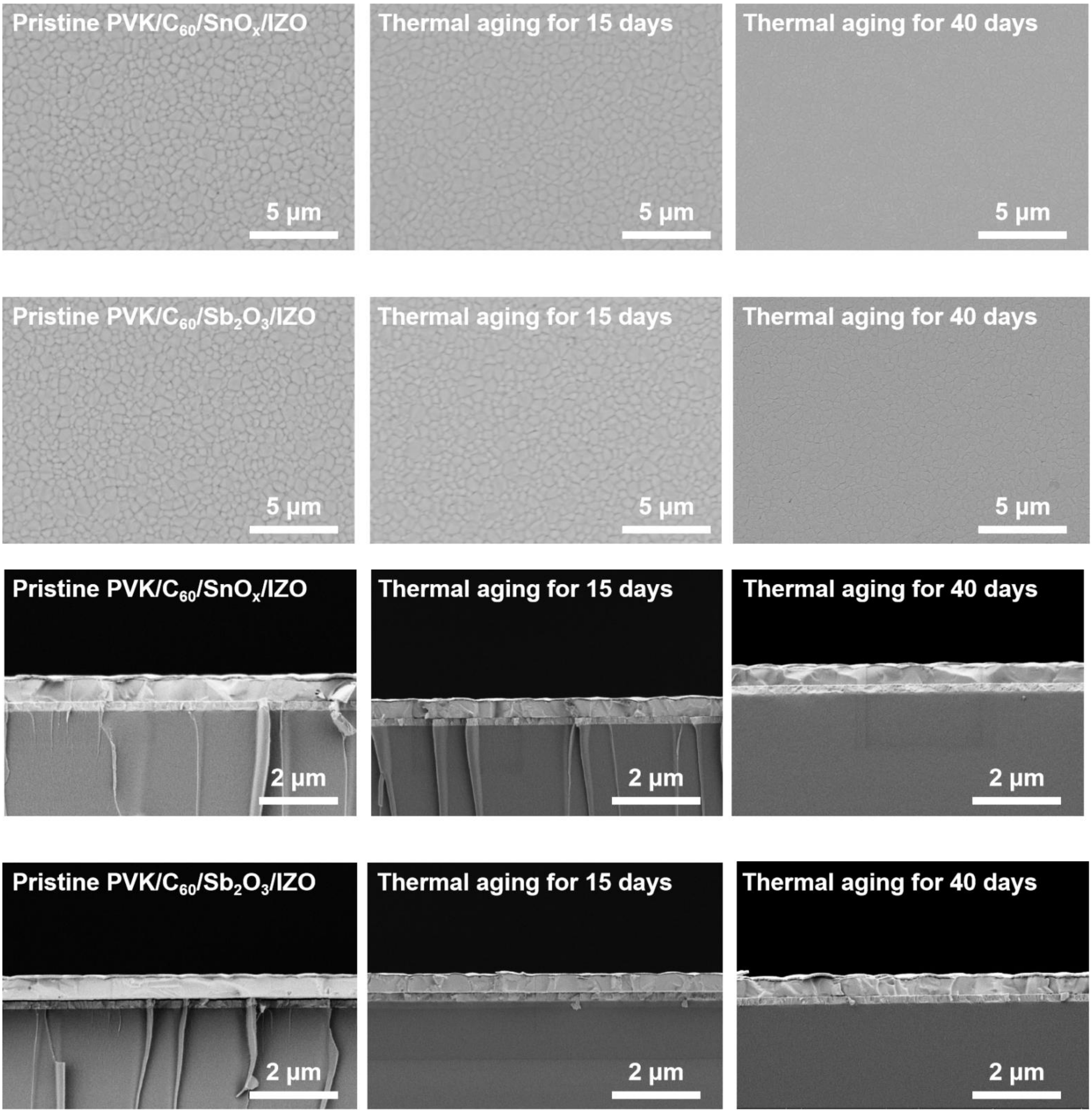

Supplementary Figure 33 SEM images of PVK/C_{60}/SnO_X or Sb_2O_3/IZO samples before and after thermal aging at 65℃ in a N_2 atmosphere.

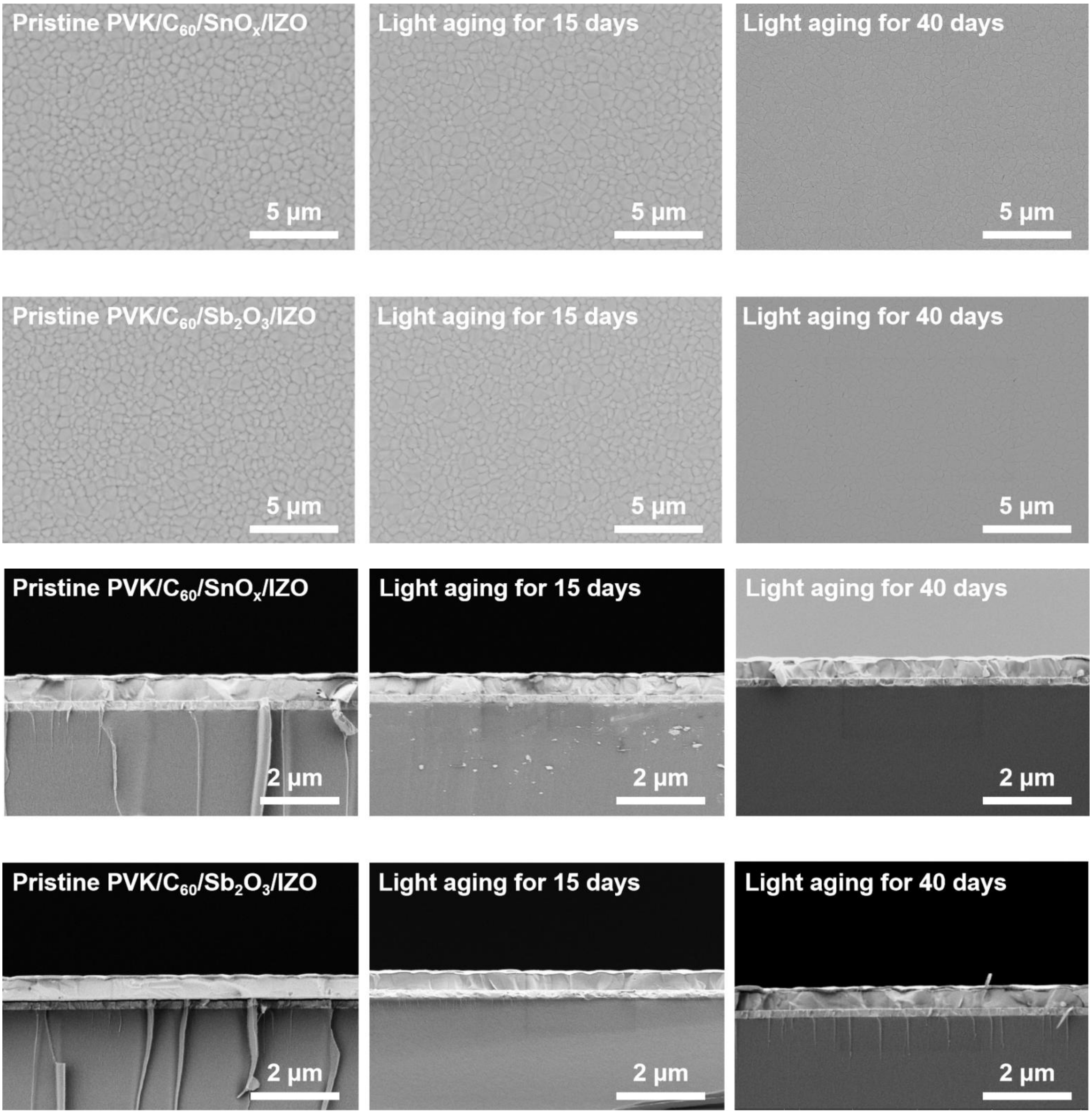

Supplementary Figure 34 SEM images of PVK/C_{60}/SnO_X or Sb_2O_3/IZO samples before and after light aging under a white LED lamp illumination at 100 mW/cm^2 in a N_2 atmosphere.

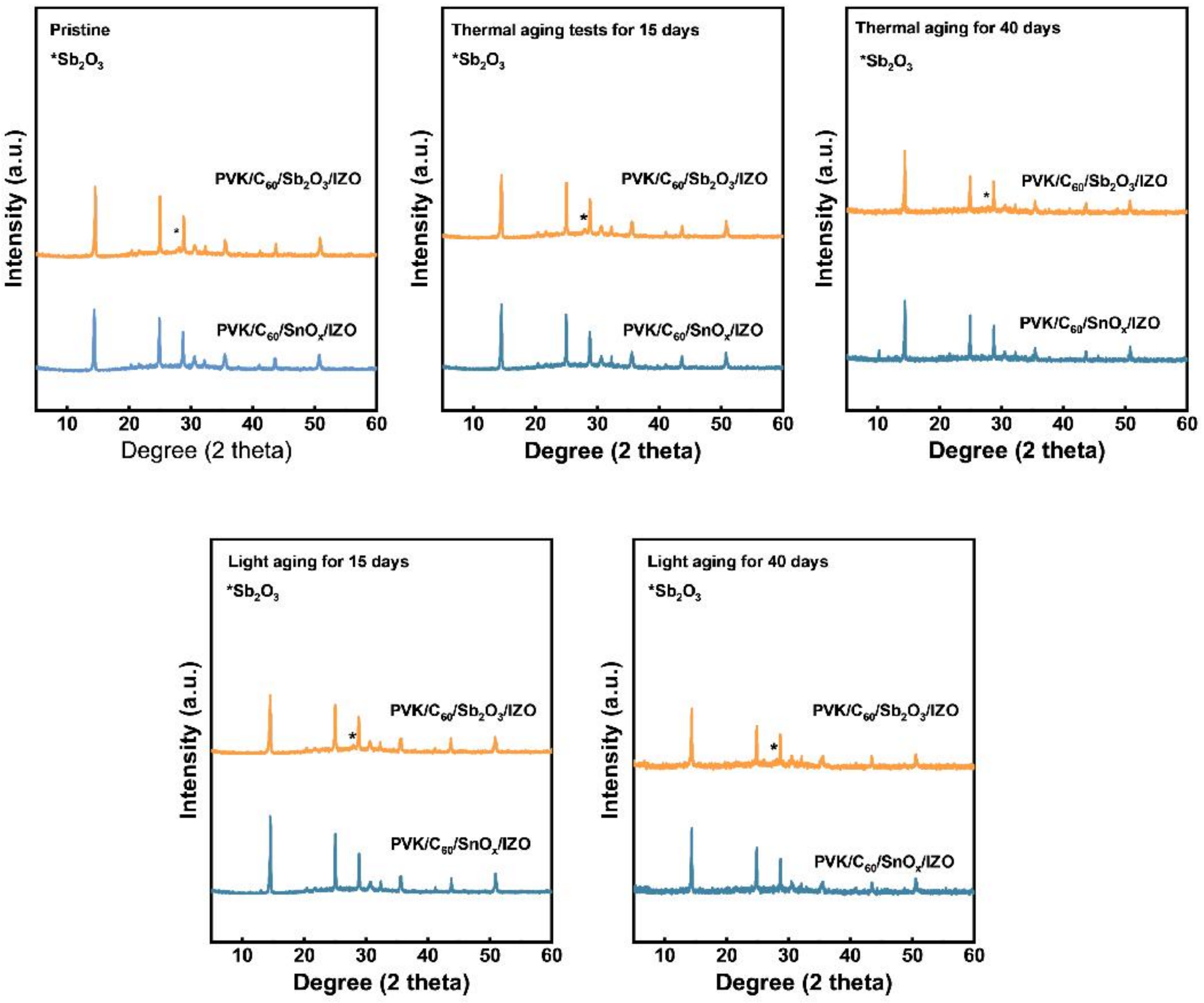

Fig .S35 XRD patterns of Glass/Me-4PACz/PVK/C_{60}/SnO_X- or Sb_2O_3/IZO stacks under different aging conditions. (i) Pristine film, and films aged after different days in N_2 atmosphere under (ii) 65 °C or (iii) a white LED source (100 mW/cm^2).

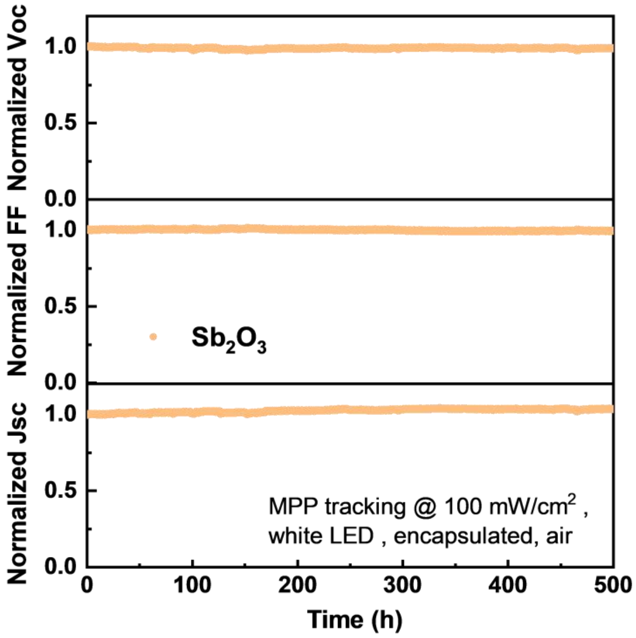

Supplementary Figure 36 Evolution of normalized V_{OC}, J_{SC}, FF of encapsulated Sb_2O_3-based PST for MPP tracking under a white LED lamp illumination at 100 mW/cm^2 in air.

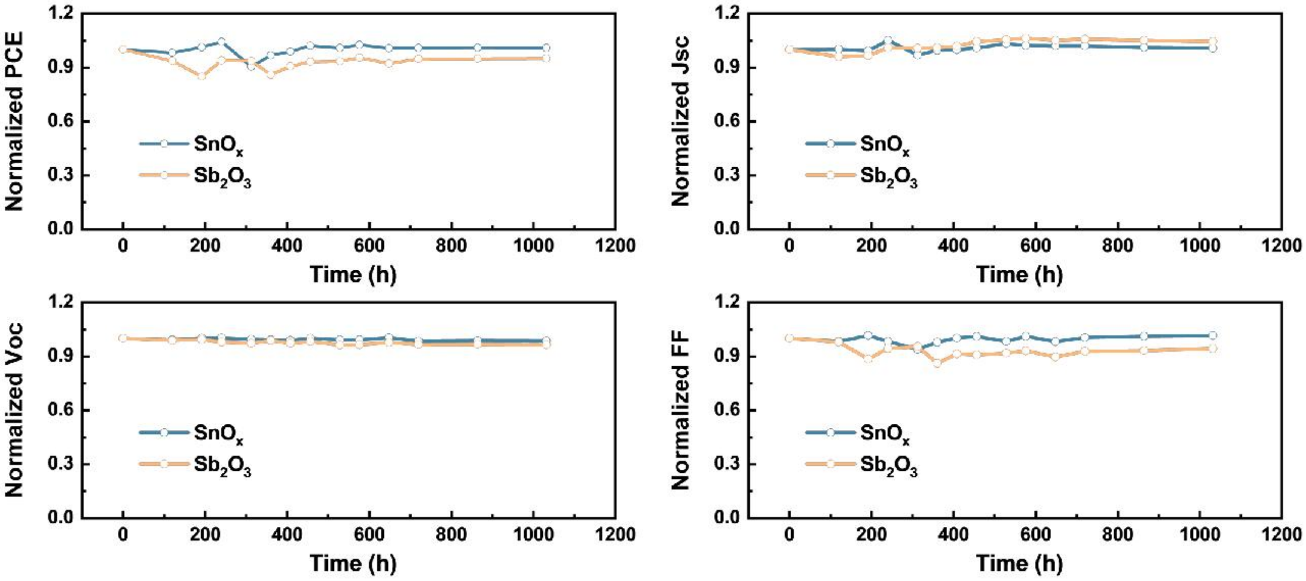

Supplementary Figure 37 Thermal aging tests of encapsulated SnO_X- or Sb_2O_3-based tandem devices at 65°C in a N_2 atmosphere.

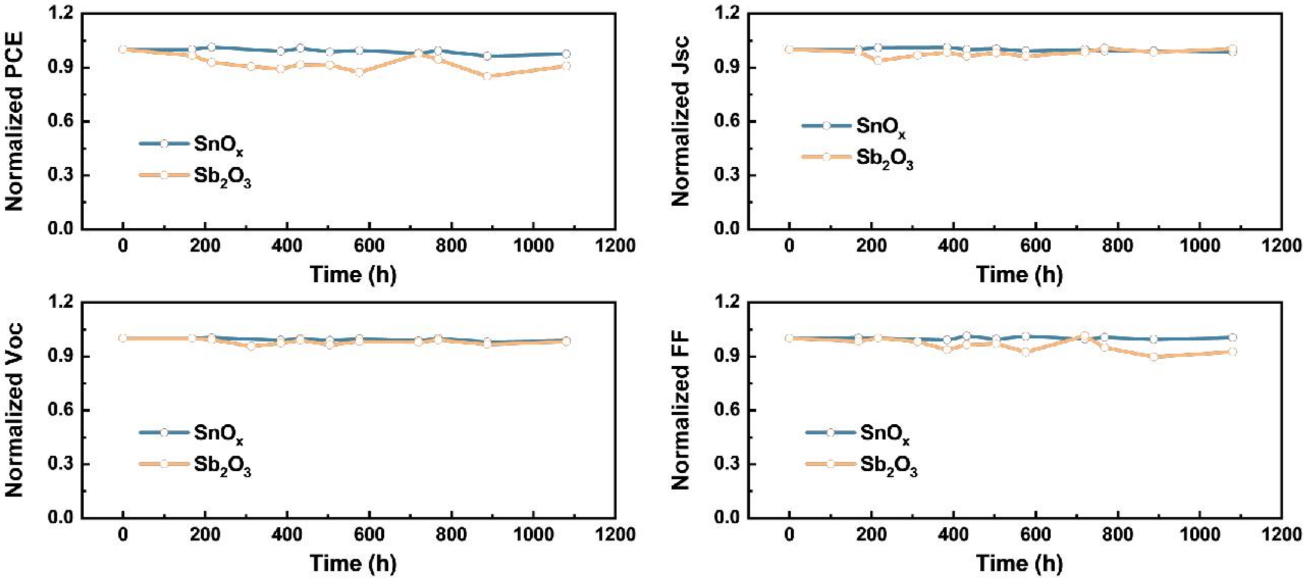

Supplementary Figure 38 Light aging tests of encapsulated SnO_X- or Sb_2O_3-based tandem devices under a white LED lamp illumination at 100 mW/cm^2 in a N_2 atmosphere.

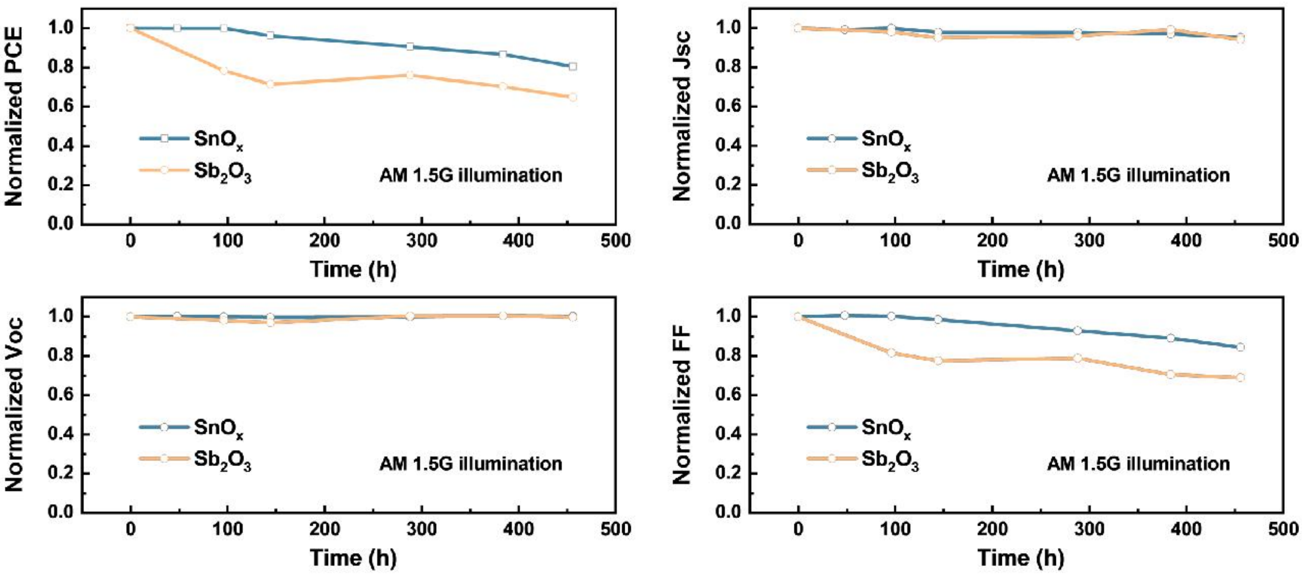

Supplementary Figure 39 Light aging tests of encapsulated SnO_X- or Sb_2O_3-based tandem devices under continuous AM 1.5G illumination at 100 mW/cm^2 in air. The light source was a xenon lamp solar simulator.

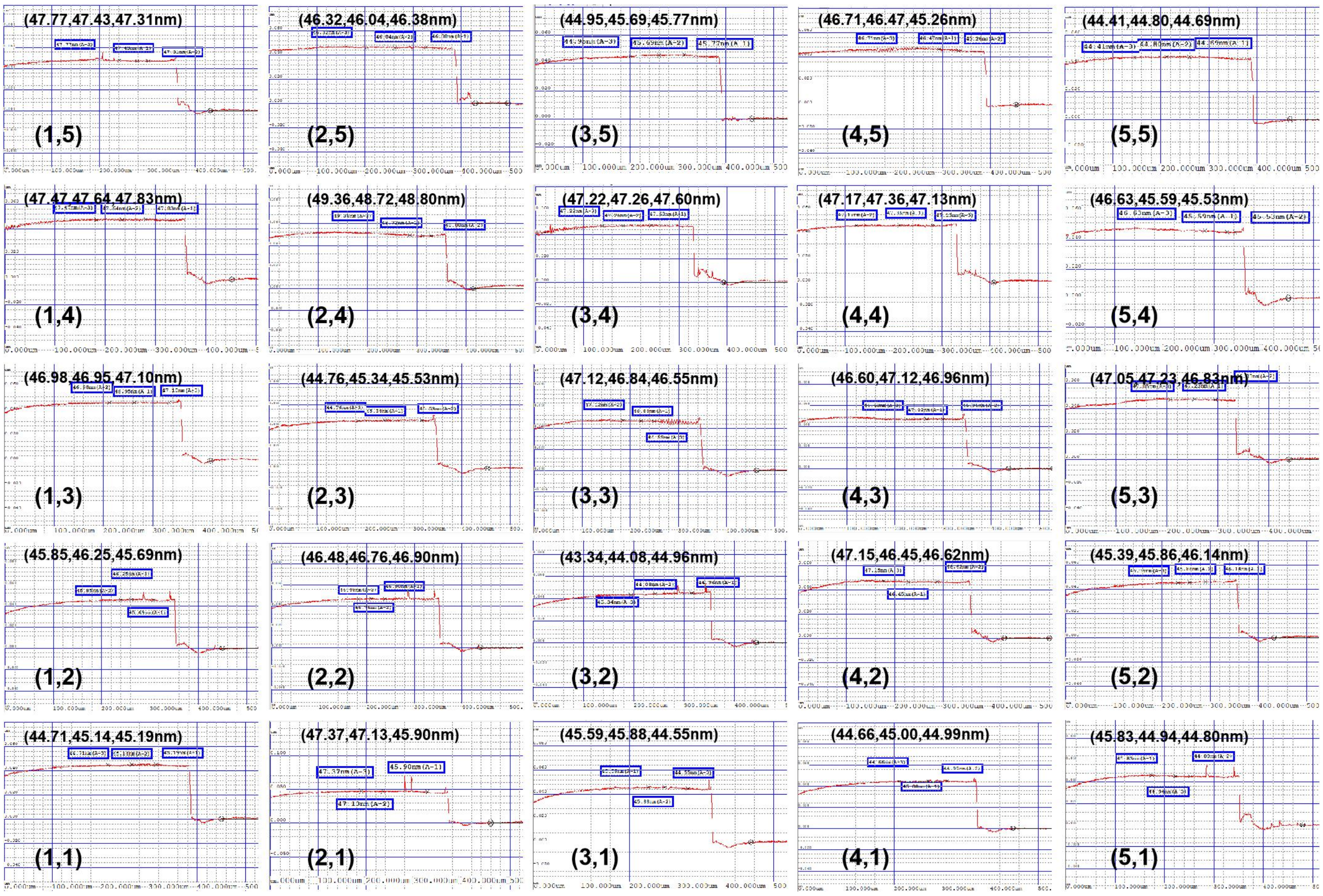

Supplementary Figure 40 Step profiler test results of Sb_2O_3 samples with different positions deposited on a 10×10 cm^2 glass substrate.

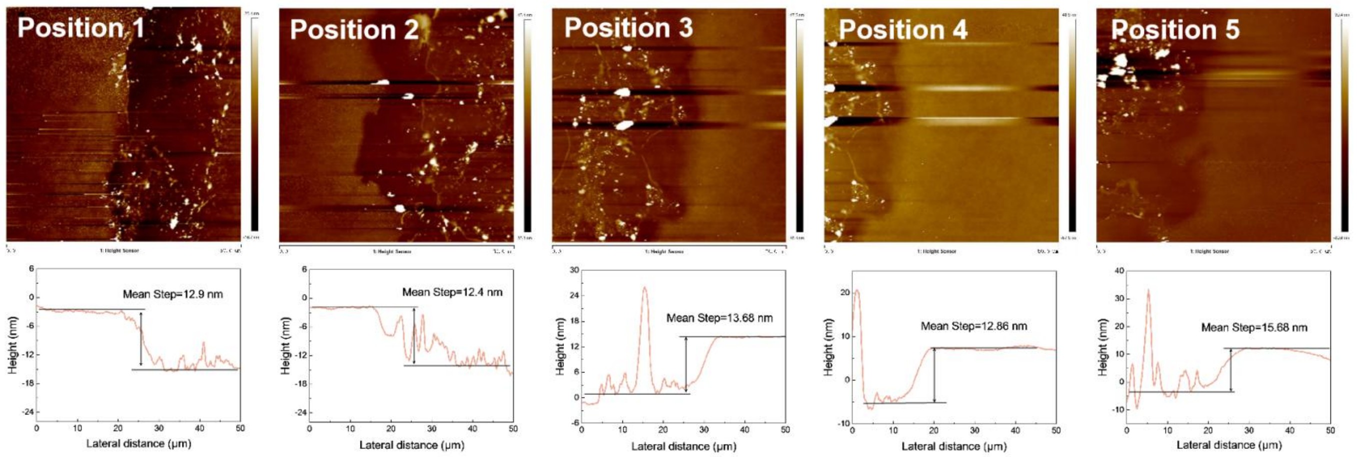

Supplementary Figure 41 AFM images and mean step height of 15 nm Sb_2O_3 films measured from five representative positions.

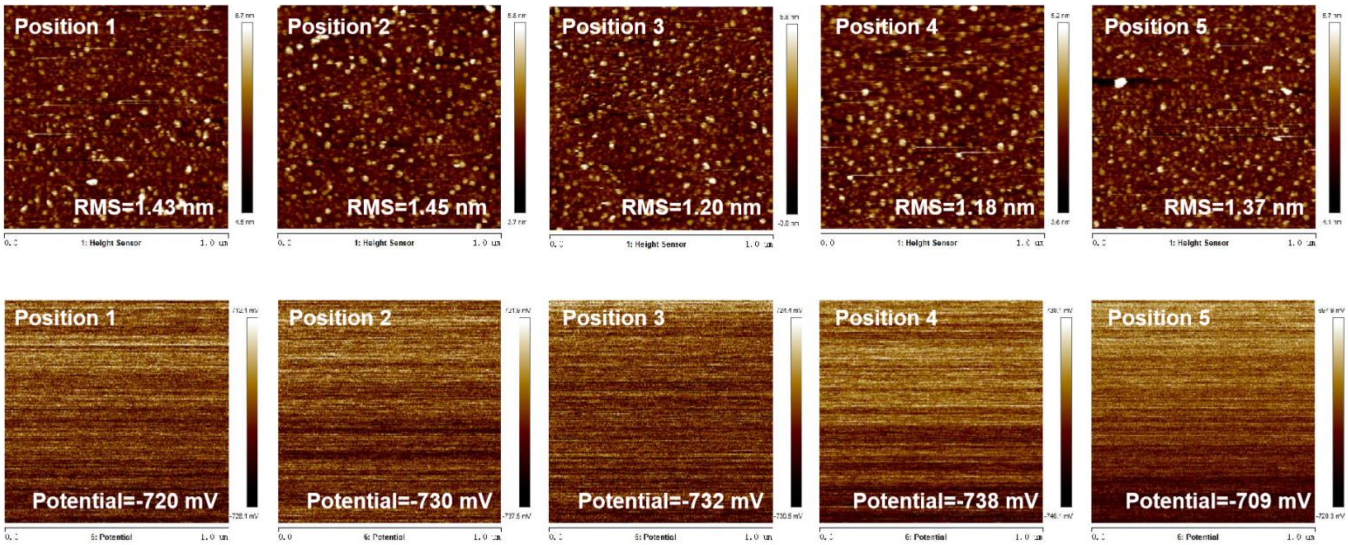

Supplementary Figure 42 AFM and corresponding kelvin probe force microscopy (KPFM) images of 15 nm Sb_2O_3 films measured from five representative positions.

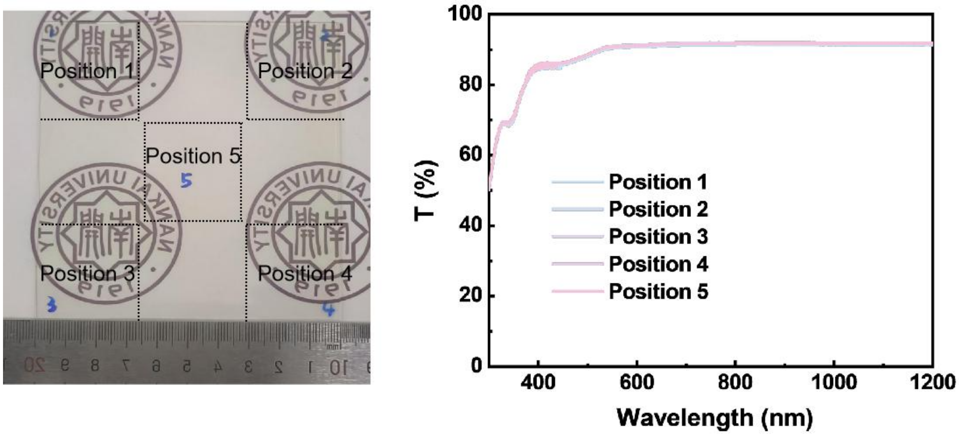

Supplementary Figure 43 Photograph of a 5 nm C_{60} on a 10×10 cm^2 glass substrate and transmittance spectra measured from five representative positions.

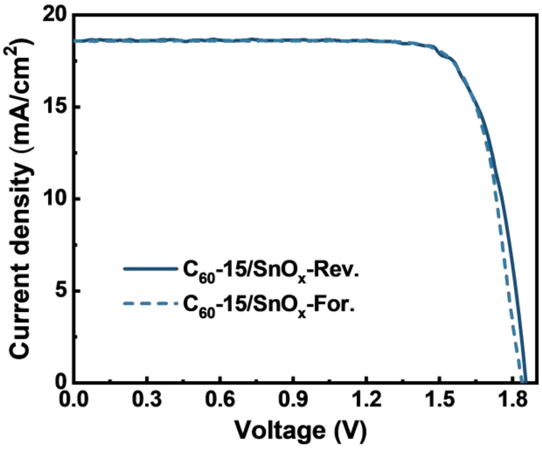

Supplementary Figure 44 The *J*-*V* curves of the encapsulated large-area champion PST (64.64 cm^2) with SnO_X.

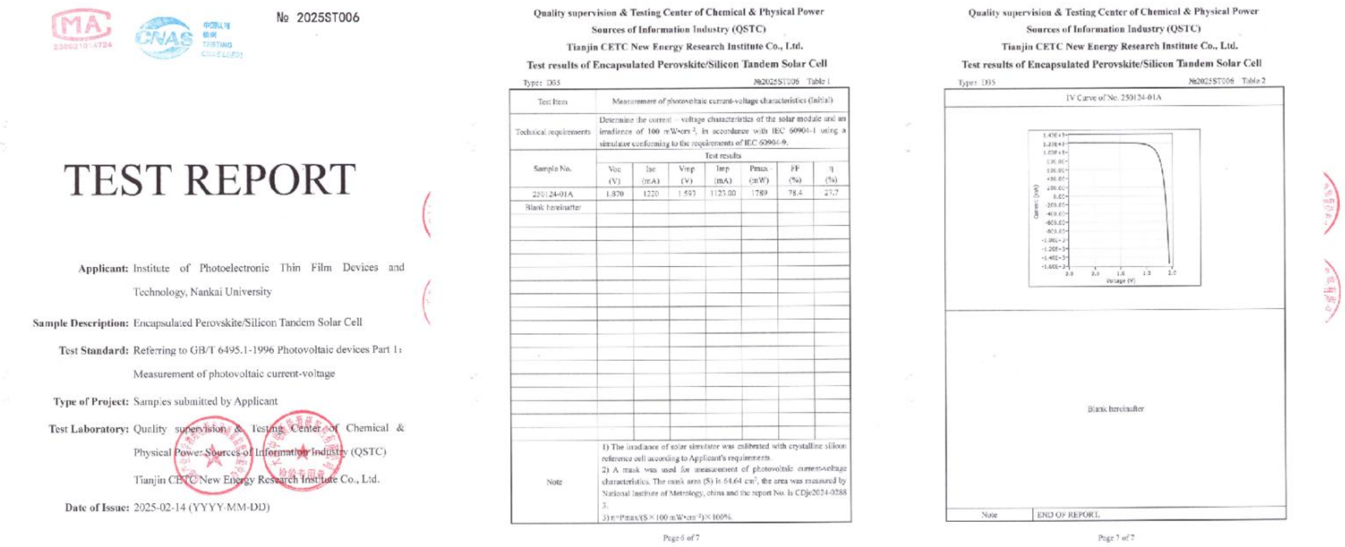
№ 2025ST006

TEST REPORT

Applicant: Institute of Photoelectronic Thin Film Devices and Technology, Nankai University

Sample Description: Encapsulated Perovskite/Silicon Tandem Solar Cell

Test Standard: Referring to GB/T 6495.1-1996 Photovoltaic devices Part 1: Measurement of photovoltaic current-voltage

Type of Project: Samples submitted by Applicant

Test Laboratory: Quality supervision & Testing Center of Chemical & Physical Power Sources of Information Industry (QSTC) Tianjin CETC New Energy Research Institute Co., Ltd.

Date of Issue: 2025-02-14 (YYYY-MM-DD)

Quality supervision & Testing Center of Chemical & Physical Power Sources of Information Industry (QSTC)
Tianjin CETC New Energy Research Institute Co., Ltd.
Test results of Encapsulated Perovskite/Silicon Tandem Solar Cell

Type: D35 №2025ST006 Table 1

Test Item	Measurement of photovoltaic current-voltage characteristics (Initial)						
Technical requirements	Determine the current – voltage characteristics of the solar module and an irradiance of 100 mW·cm⁻², in accordance with IEC 60904-1 using a simulator conforming to the requirements of IEC 60904-9.						
Sample No.	Test results						
	Voc (V)	Isc (mA)	Vmp (V)	Imp (mA)	Pmax (mW)	FF (%)	η (%)
250124-01A	1.870	1220	1.593	1123.00	1789	78.4	27.7
Blank hereinafter							
Note	1) The irradiance of solar simulator was calibrated with crystalline silicon reference cell according to Applicant's requirements. 2) A mask was used for measurement of photovoltaic current-voltage characteristics. The mask area (S) is 64.64 cm², the area was measured by National Institute of Metrology, china and the report No. is CDjc2024-02883. 3) η=Pmax/(S×100 mW·cm⁻²)×100%.						

Page 6 of 7

Quality supervision & Testing Center of Chemical & Physical Power Sources of Information Industry (QSTC)
Tianjin CETC New Energy Research Institute Co., Ltd.
Test results of Encapsulated Perovskite/Silicon Tandem Solar Cell

Type: D35 №2025ST006 Table 2

IV Curve of No. 250124-01A

Blank hereinafter

Note: END OF REPORT.

Page 7 of 7

Supplementary Figure 45 The certification result of encapsulated large-area PSTs (aperture area of 64.64 cm^2) at CETC. The reverse-scan delivered a PCE of 27.7%, with a J_{SC} of 18.87 mA/cm^2, V_{OC} of 1.870 V, and FF of 78.4%.

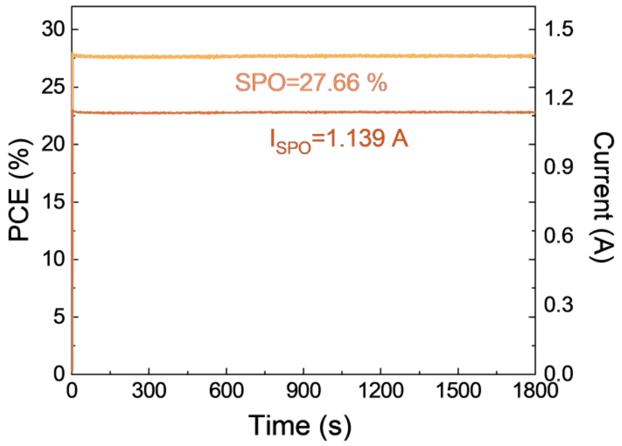

Supplementary Figure 46 Stabilized power output (SPO) of the encapsulated large-area champion PST (64.64 cm^2) with Sb_2O_3.

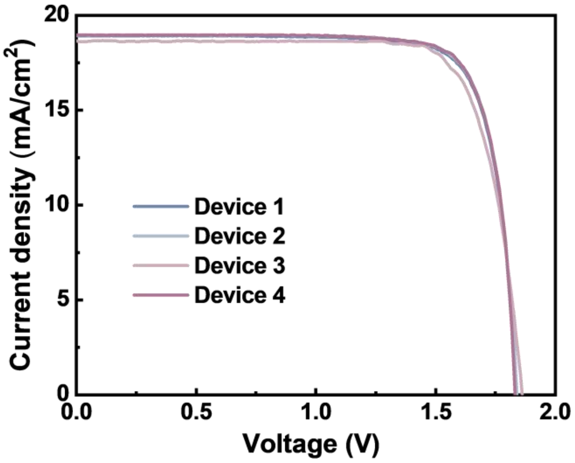

Supplementary Figure 47 *J–V* curves of four large-area (64.64 cm^2) devices from different fabrication batches.

Supplementary Tables

Supplementary Table 1 Melting points of various metal oxides.

Metal oxides	ZnO	TiO_2	SnO_2	GeO_2	Sb_2O_3
Melting point (°C)	1975	1840	1630	1115	655

Supplementary Table 2 PV parameters of single-junction PSCs with an aperture 0.0755 cm^2.

	V_{OC} (V)	J_{SC} (mA/cm^2)	FF (%)	PCE (%)
SnO_X-1.64 eV	1.228	22.48	83.92	23.17
Sb_2O_3-1.59 eV	1.173	22.70	83.69	22.28
Sb_2O_3-1.62 eV	1.210	21.92	84.88	22.50
Sb_2O_3-1.64 eV	1.227	22.34	84.55	23.18
Sb_2O_3-1.68 eV	1.214	21.76	84.40	22.29

Supplementary Table 3 Published performances of single junction mid/wide-bandgap bandgap (1.61-1.68 eV) PSCs prepared via a vacuum-solution hybrid deposition.

Device Structure	E_g (eV)	V_{OC} (V)	J_{SC} (mA/cm^2)	FF (%)	PCE (%)	Ref.
Glass/ITO/Spiro-TTB/PVK/C_{60}/ZnO/Al	1.65	1.030	17.70	78.35	15.25	21
Glass/FTO/TiO_2/PCBM/PVK/Spiro-MoTAD/Au	1.60	1.103	22.70	74.50	18.90	22
Glass/ITO/Me-4PACz/PVK/C_{60}/BCP or SnO_X/Au or Ag	1.70	-	-	-	19.50	23
Glass/ITO/MoO_X/TaTm/PVK/C_{60}/BCP/Ag	1.65	1.150	20.80	82.80	19.70	24
Glass/ITO/Spiro-TTB/PVK/C_{60}/ZnO/Ag	1.62	1.112	21.31	83.56	19.80	25
Glass/FTO/SnO_2/PVK/Spiro-MoTAD/Au	1.64	1.140	22.00	79.00	19.80	26
Glass/ITO/Me-2PACz/PVK/C_{60}/BCP/Cu	1.61	1.160	21.84	79.96	20.26	27
Glass/ITO/NiO_X/SAMs/PVK/C_{60}/BCP/Cu	1.68	1.207	20.90	80.80	20.30	28
MgF_2/Glass/ITO/NiO_X/PVK/LiF/C_{60}/BCP/Cu	1.67	1.140	22.06	80.88	20.34	29
Glass/ITO/NiO_X/Me-2PACz/PVK/C_{60}/BCP/Ag	1.65	1.130	22.77	80.62	20.62	30
Glass/ITO/Spiro-TTB/PVK/C_{60}/BCP/Ag	1.61	1.188	21.63	80.72	20.74	31
Glass/ITO/NiO_X/SAMs/PVK/C_{60}/SnO_X/Cu	1.68	1.220	20.98	81.46	20.77	32
Glass/ITO/NiO_X/SAMs/PVK/C_{60}/SnO_X/Ag	1.68	1.190	20.90	81.60	20.90	33
Glass/ITO/NiO_X/2PACz/PVK/LiF/C_{60}/BCP/Ag	1.65	1.160	22.98	78.97	21.05	34
MgF_2/Glass/ITO/NiO_X/PVK/LiF/C_{60}/BCP/Cu	1.64	1.110	23.90	79.50	21.06	29
Glass/ITO/NiO_X/2PACz/PVK/LiF/C_{60}/BCP/Ag	1.65	1.140	23.19	80.60	21.31	35
Glass/ITO/Spiro-TTB/PVK/C_{60}/BCP/Ag	1.60	1.150	22.77	85.48	22.74	36
Glass/ITO/Me-4PACz/PVK/C_{60}/Sb_2O_3/Al	1.59	1.173	22.70	83.69	22.28	This work
Glass/ITO/Me-4PACz/PVK/C_{60}/Sb_2O_3/Al	1.62	1.210	21.92	84.88	22.50	This work
Glass/ITO/Me-4PACz/PVK/C_{60}/Sb_2O_3/Al	1.64	1.227	22.34	84.55	23.18	This work
Glass/ITO/Me-4PACz/PVK/C_{60}/Sb_2O_3/Al	1.68	1.214	21.76	84.40	22.29	This work

Supplementary Table 4 PV parameters of champion PSTs with an aperture of 1 cm^2.

	Scanning direction	V_{OC} (V)	J_{SC} (mA/cm^2)	FF (%)	PCE (%)
C_{60}-15/SnO_X	Rev.	1.897	19.22	78.43	28.59
	For.	1.884	19.26	75.70	27.46
C_{60}-5/Sb_2O_3	Rev.	1.900	20.26	78.67	30.28
	For.	1.884	20.25	76.24	29.08

Supplementary Table 5 The average transmittance of 5 nm C_{60} film in the wavelength range from 300 nm to 560 nm measured from five representative positions.

	Position 1	Position 2	Position 3	Position 4	Position 5
Average transmittance (%)	81.50	81.78	81.84	81.97	82.08

Supplementary Table 6 PV parameters of encapsulated champion PSTs with an aperture of 64.64 cm^2.

	Scanning direction	V_{OC} (V)	J_{SC} (mA/cm^2)	FF (%)	PCE (%)
SnO_X	Rev.	1.856	18.61	79.02	27.29
	For.	1.839	18.59	79.55	27.20
Sb_2O_3	Rev.	1.832	18.96	81.11	28.16
	For.	1.821	19.01	80.68	27.93

Supplementary Table 7 Published performances of large-area champion PSTs (>10 cm^2).

Device Structure	Area (cm^2)	V_{OC} (V)	J_{SC} (mA/cm^2)	FF (%)	PCE (%)	Ref.
PERC/SnO_2/PVK/Spiro-OMeTAD/MoO_X/ITO/Au	25	1.784	14.40	67.00	17.30	37
PERC/SnO_2/PVK/Spiro-OMeTAD/MoO_X/ITO/Ag/AR foil	16	1.658	15.60	68.00	17.60	38
SHJ/nc-Si:H (p^+)/nc-Si:H (n^+)/PVK/Spiro-OMeTAD/MoO_X/IZO/Au/MgF_2	12.96	1.769	16.50	65.40	19.10	39
SHJ/SnO_2/PVK/Spiro-OMeTAD/MoO_3/ITO/Ag/PDMS	65.1	1.777	17.60	67.50	21.10	40
SHJ/ITO/NiO_X/PVK/C_{60}/SnO_2/ITO/Ag/MgF_2	57.4	1.723	17.48	75.00	22.60	41
SHJ/ITO/NiO_X/Poly-TPD/PVK/C_{60}/SnO_X/IZO/Ag/MgF_2	14.44	1.915	16.15	78.30	24.22	42
SHJ/ITO/PTAA/LiF/PVK/C_{60}/SnO_2/ITO/Ag/PDMS	24	1.890	18.10	73.60	25.20	43
SHJ/nc-Si:H (n^+)/nc-Si:H (p^+)/Spiro-TTB/PVK/C_{60}/SnO_2/IZO/Al/MgF_2	11.879	1.852	18.11	74.93	25.13 (24.34)*	44
SHJ/nc-Si:H (n^+)/nc-Si:H (p^+)/Spiro-TTB/PVK/C_{60}/SnO_2/IZO/Al/MgF_2	11.25	1.872	18.15	74.85	25.43	25
SHJ/ITO/NiO_X/SAM/PVK/C_{60}/SnO_X/IZO/Ag/MgF_2	16	1.815	18.54	78.31	26.30	32
SHJ/ITO/Poly-TPD/PVK/C_{60}/SnO_2/ITO/Ag/MgF_2	25	1.940	19.20	79.00	29.40 (28.80)*	45
SHJ/IZO/NiO_X/Me-4PACz/PVK/C_{60}/Sb_2O_3/IZO/Ag/MgF_2	64.64	1.832	19.02	81.11	28.16 (27.70)*	This work

*Certified PCE.

Table. S8 PV parameters of four large-area (64.64 cm^2) devices from different fabrication batches.

	V_{OC} (V)	J_{SC} (mA/cm^2)	FF (%)	PCE (%)
Device 1	1.839	18.93	80.02	27.86
Device 2	1.840	18.95	80.42	28.04
Device 3	1.862	18.66	78.31	27.21
Device 4	1.832	18.96	81.11	28.16
Average	1.843	18.88	79.97	27.82
Std. Dev.	0.0130	0.1439	1.192	0.4233
RSD	0.71%	0.76%	1.49%	1.52%

Supplementary Table 9 Fabrication cost comparison between C_{60}-15/SnO_X and C_{60}-5/Sb_2O_3 stacks for 100 cm^2 PSTs.

	Common combination		Innovation combination	
	C_{60}	SnO_X	C_{60}	Sb_2O_3
Layer thickness per device (nm)	15	15	5	15
Film volume for 100 cm^2 (cm^3)	0.00015	0.00015	0.00005	0.00015
Film density (g/cm^3)	1.65[46]	6.90[47]	1.65	5.7[48]
Film quality (g)	0.0002475	0.001035	0.0000825	0.000855
Average material utilization factor [a]	0.1	0.04	0.1	0.06
Usage of precursor (g)	0.002475	0.025875	0.000825	0.01425
Unit price of material[b] (CNY/g)	800	100	800	41[49]
Material Cost (RMB/100 cm^2)	1.98	2.5875	0.66	0.58425
**Total Material Cost (RMB/100 cm^2)**	4.5675		1.24425	
Equipment investment (CNY)[b]	394000	360000	394000	
Equipment lifetime (h)	72000	72000	72000	
Equipment depreciation cost (CNY/h)	5.5	5	5.5	
Processing duration (h)[c]	1	2.5	1.1	
Equipment depreciation cost (CNY/100 cm^2)	5.5	12.5	6.05	
**Total equipment depreciation cost (CNY/100 cm^2)**	18		6.05	
Equipment power (KW)	6	8	6	
Processing duration (h)	1	2.5	1.1	
Average electricity price (CNY/KW/h)	0.52	0.52	0.52	
Electricity cost (CNY/100 cm^2)	3.12	10.4	3.432	
**Total electricity cost**	13.52		3.432	
**Total cost (CNY/100 cm^2)**	36.09		10.73	

Supplementary References

1 Yu, Z. *et al.* Simplified interconnection structure based on C_{60}/SnO_{2-x} for all-perovskite tandem solar cells. *Nature Energy* **5**, 657-665 (2020). https://doi.org/10.1038/s41560-020-0657-y

2 Haj Lakhdar, M., Smida, Y. B. & Amlouk, M. Synthesis, optical characterization and DFT calculations of electronic structure of Sb_2O_3 films obtained by thermal oxidation of Sb_2S_3. *Journal of Alloys and Compounds* **681**, 197-204 (2016). https://doi.org/10.1016/j.jallcom.2016.04.026

3 Chen, P. *et al.* Multifunctional ytterbium oxide buffer for perovskite solar cells. *Nature* **625**, 516-522 (2024). https://doi.org/10.1038/s41586-023-06892-x

4 Kresse, G. & Furthmüller, J. Efficiency of ab-initio total energy calculations for metals and semiconductors using a plane-wave basis set. *Computational materials science* **6**, 15-50 (1996). https://doi.org/https://doi.org/10.1016/0927-0256(96)00008-0

5 Blöchl, P. E. Projector augmented-wave method. *Physical review B* **50**, 17953 (1994). https://doi.org/https://doi.org/10.1103/PhysRevB.50.17953

6 Ning, J. *et al.* Workhorse minimally empirical dispersion-corrected density functional with tests for weakly bound systems: r^2SCAN+rVV10. *Physical Review B* **106** (2022). https://doi.org/10.1103/PhysRevB.106.075422

7 Liu, K. *et al.* A wafer-scale van der Waals dielectric made from an inorganic molecular crystal film. *Nature Electronics* **4**, 906-913 (2021). https://doi.org/10.1038/s41928-021-00683-w

8 Tran, F. & Blaha, P. Accurate band gaps of semiconductors and insulators with a semilocal exchange-correlation potential. *Physical Review Letters* **102** (2009). https://doi.org/10.1103/PhysRevLett.102.226401

9 Zheng, L. *et al.* Strain-induced rubidium incorporation into wide-bandgap perovskites reduces photovoltage loss. *Science* **388**, 88-95 (2025). https://doi.org/10.1126/science.adt3417

10 Liang, Z. *et al.* Homogenizing out-of-plane cation composition in perovskite solar cells. *Nature* **624**, 557-563 (2023). https://doi.org/10.1038/s41586-023-06784-0

11 Palmstrom, A. F. *et al.* Interfacial effects of tin oxide atomic layer deposition in metal halide perovskite photovoltaics. *Advanced Energy Materials* **8**, 1800591 (2018). https://doi.org/10.1002/aenm.201800591

12 Hultqvist, A. *et al.* SnOx atomic layer deposition on bare perovskite—an investigation of initial growth dynamics, interface chemistry, and solar cell performance. *ACS Applied Energy Materials* **4**, 510-522 (2021). https://doi.org/10.1021/acsaem.0c02405

13 Zhou, B., Zhou, W. & Wu, P. Ferromagnetic ordering and metallic-like conductivity in sputtered SnNx films. *Journal of Alloys and Compounds* **604**, 106-111 (2014). https://doi.org/10.1016/j.jallcom.2014.03.098

14 Guo, H. *et al.* Immobilizing surface halide in perovskite solar cells via calix [4] pyrrole. *Advanced Materials* **35**, 2301871 (2023). https://doi.org/10.1002/adma.202301871

15 Wu, W. *et al.* Stable and uniform self-assembled organic diradical molecules for perovskite photovoltaics. *Science* **387**, eadv4551 (2025). https://doi.org/10.1126/science.adv4551

16 Jia, L. *et al.* Efficient perovskite/silicon tandem with asymmetric self-assembly molecule. *Nature* **644**, 912-919 (2025). https://doi.org/10.1038/s41586-025-09333-z

17 Liu, J. *et al.* Perovskite/silicon tandem solar cells with bilayer interface passivation. *Nature* **635**, 596-603 (2024). https://doi.org/10.1038/s41586-024-07997-7

18 Zafoschnig, L. A., Nold, S. & Goldschmidt, J. C. The race for lowest costs of electricity production: techno-economic analysis of silicon, perovskite and tandem solar cells. *IEEE Journal of Photovoltaics* **10**, 1632-1641 (2020). https://doi.org/10.1109/jphotov.2020.3024739

19 Chang, N. L. *et al.* A bottom-up cost analysis of silicon–perovskite tandem photovoltaics. *Progress in Photovoltaics: Research and Applications* **29**, 401-413 (2020). https://doi.org/10.1002/pip.3354

20 Cordell, J. J., Woodhouse, M. & Warren, E. L. Technoeconomic analysis of perovskite/silicon tandem solar modules. *Joule* **9** (2025). https://doi.org/10.1016/j.joule.2024.10.013
21 Li, Y. *et al.* Wide bandgap interface layer induced stabilized perovskite/silicon tandem solar cells with stability over ten thousand hours. *Advanced Energy Materials* **11**, 2102046 (2021). https://doi.org/10.1002/aenm.202102046
22 Rafizadeh, S. *et al.* Efficiency enhancement and hysteresis mitigation by manipulation of grain growth conditions in hybrid evaporated–spin-coated perovskite solar cells. *ACS Applied Materials & Interfaces* **11**, 722-729 (2018). https://doi.org/10.1021/acsami.8b16963
23 Chin, X. Y. *et al.* Interface passivation for 31.25%-efficient perovskite/silicon tandem solar cells. *Science* **381**, 59-62 (2023). https://doi.org/10.1126/science.adg0091
24 Nguyen, V. S. *et al.* Solvent-vapor assisted conversion process for hybrid perovskites coupling thermal evaporation and slot-die coating. *Materials Science in Semiconductor Processing* **158**, 107358 (2023). https://doi.org/10.1016/j.mssp.2023.107358
25 Li, Y. *et al.* CsCl induced efficient fully-textured perovskite/crystalline silicon tandem solar cell. *Nano Energy* **122**, 109285 (2024). https://doi.org/10.1016/j.nanoen.2024.109285
26 Soltanpoor, W. *et al.* Hybrid vapor-solution sequentially deposited mixed-halide perovskite solar cells. *ACS Applied Energy Materials* **3**, 8257-8265 (2020). https://doi.org/10.1021/acsaem.0c00686
27 Zhang, F. *et al.* Buried-interface engineering of conformal 2D/3D perovskite heterojunction for efficient perovskite/silicon tandem solar cells on industrially textured silicon. *Advanced Materials* **35**, 2303139 (2023). https://doi.org/10.1002/adma.202303139
28 Luo, H. *et al.* Inorganic framework composition engineering for scalable fabrication of perovskite/silicon tandem solar cells. *ACS Energy Letters* **8**, 4993-5002 (2023). https://doi.org/10.1021/acsenergylett.3c02002
29 Afshord, A. Z. *et al.* Efficient and stable inverted wide-bandgap perovskite solar cells and modules enabled by hybrid evaporation-solution method. *Advanced Functional Materials* **33**, 2301695 (2023). https://doi.org/10.1002/adfm.202301695
30 Sun, Y. *et al.* Ionic liquid modified polymer intermediate layer for improved charge extraction toward efficient and stable perovskite/silicon tandem solar cells. *Small* **20**, 2308553 (2024). https://doi.org/10.1002/smll.202308553
31 Xu, Q. *et al.* Diffusible capping layer enabled homogeneous crystallization and component distribution of hybrid sequential deposited perovskite. *Advanced Materials* **36**, 2308692 (2024). https://doi.org/10.1002/adma.202308692
32 Zheng, X. *et al.* Solvent engineering for scalable fabrication of perovskite/silicon tandem solar cells in air. *Nature Communications* **15**, 4907 (2024). https://doi.org/10.1038/s41467-024-49351-5
33 Luo, X. *et al.* Efficient perovskite/silicon tandem solar cells on industrially compatible textured silicon. *Advanced Materials* **35**, 2207883 (2023). https://doi.org/10.1002/adma.202207883
34 Yang, T. *et al.* Efficient and stable perovskite/silicon tandem solar cells modulated with triple-functional passivator. *Advanced Energy Materials* **14**, 2303149 (2024). https://doi.org/10.1002/aenm.202303149
35 Mao, L. *et al.* Fully textured, production-line compatible monolithic perovskite/silicon tandem solar cells approaching 29% efficiency. *Advanced Materials* **34**, 2206193 (2022). https://doi.org/10.1002/adma.202206193
36 Liu, J. *et al.* Textured perovskite/silicon tandem solar cells achieving over 30% efficiency promoted by 4-fluorobenzylamine hydroiodide. *Nano-Micro Letters* **16**, 189 (2024). https://doi.org/10.1007/s40820-024-01406-4
37 Hyun, J. Y. *et al.* Perovskite/Silicon Tandem Solar Cells with a V_{oc} of 1784 mV Based on an Industrially Feasible 25 cm^2 TOPCon Silicon Cell. *ACS Applied Energy Materials* **5**, 5449-5456 (2022). https://doi.org/10.1021/acsaem.1c02796

38 Zheng, J. *et al.* Large area efficient interface layer free monolithic perovskite/homo-junction-silicon tandem solar cell with over 20% efficiency. *Energy & Environmental Science* **11**, 2432-2443 (2018). https://doi.org/10.1039/c8ee00689j
39 Sahli, F. *et al.* Improved optics in monolithic perovskite/silicon tandem solar cells with a nanocrystalline silicon recombination junction. *Advanced Energy Materials* **8**, 1701609 (2018). https://doi.org/10.1002/aenm.201701609
40 Zheng, J. *et al.* Efficient monolithic perovskite–Si tandem solar cells enabled by an ultra-thin indium tin oxide interlayer. *Energy & Environmental Science* **16**, 1223-1233 (2023). https://doi.org/10.1039/d2ee04007g
41 Kamino, B. A. *et al.* Low-temperature screen-printed metallization for the scale-up of two-terminal perovskite–silicon tandems. *ACS Applied Energy Materials* **2**, 3815-3821 (2019). https://doi.org/10.1021/acsaem.9b00502
42 Qiang, Z. *et al.* A scalable method for fabricating monolithic perovskite/silicon tandem solar cells based on low-cost industrial silicon bottom cells. *Chemical Engineering Journal* **495**, 153422 (2024). https://doi.org/10.1016/j.cej.2024.153422
43 Yang, G. *et al.* Shunt mitigation toward efficient large-area perovskite-silicon tandem solar cells. *Cell Reports Physical Science* **4**, 101628 (2023). https://doi.org/10.1016/j.xcrp.2023.101628
44 Xu, Q. *et al.* Conductive passivator for efficient monolithic perovskite/silicon tandem solar cell on commercially textured silicon. *Advanced Energy Materials* **12**, 2202404 (2022). https://doi.org/10.1002/aenm.202202404
45 Chen, Y. *et al.* Nuclei engineering for even halide distribution in stable perovskite/silicon tandem solar cells. *Science* **385**, 554-560 (2024). https://doi.org/10.1126/science.ado9104
46 MatWeb. Carbon (fullerene-C_{60}). https://matweb.com/search/DataSheet.aspx?MatGUID=079e7b90a5914e24bc272fbb0e15fa9b&ckck=1 (2025).
47 MatWeb. Tin oxide nanopowder. https://matweb.com/search/DataSheet.aspx?MatGUID=8161a17bd90f4ec2b2a16fe15db6c056 (2025).
48 MatWeb. Antimony oxide, Sb_2O_3 (Valentinite). https://matweb.com/search/DataSheet.aspx?MatGUID=7fd3759d5bed4c5b9b832ef8faff6dad (2025).
49 Sigma-Aldrich. Antimony oxide (Cat. No. 379255). https://www.sigmaaldrich.cn/CN/zh/product/aldrich/379255 (2025).
