# Supplementary material for: Antimony oxide buffer layer for single- and double-junction perovskite-based solar cells
Source: Nat Commun. 2026 Mar 25;17:4394. doi: 10.1038/s41467-026-70848-8 (PMC13181109; doi:10.1038/s41467-026-70848-8)
Supplement: Supplementary file 2 — Reporting Summary [file 41467_2026_70848_MOESM2_ESM.pdf]

nature portfolio

Corresponding author(s): Xiaodan Zhang, Stefaan De Wolf, Yongcai He, Lifan Gao

Solar Cells Reporting Summary

Nature Portfolio wishes to improve the reproducibility of the work that we publish. This form is intended for publication with all accepted papers reporting the characterization of photovoltaic devices and provides structure for consistency and transparency in reporting. Some list items might not apply to an individual manuscript, but all fields must be completed for clarity.

For further information on Nature Research policies, including our data availability policy, see Authors & Referees.

Experimental design

Please check the following details are reported in the manuscript, and provide a brief description or explanation where applicable.

1. Dimensions

Area of the tested solar cells

- [x] Yes
- [ ] No

0.0755 cm2 aperture area for single-junction solar cell and 1 cm2, 64.64 cm2 aperture area for tandem solar cells, respectively.

Explain why this information is not reported/not relevant.

Method used to determine the device area

- [ ] Yes
- [x] No

Provide a description of the method and state where this information can be found in the text.

The area of the solar cells tested is defined by a non-reflective metal mask with an aperture area of 0.0755 cm2, 1 cm2 and 64.64 cm2 for regular measured by measuring microscope.

2. Current-voltage characterization

Current density-voltage (J-V) plots in both forward and backward direction

- [x] Yes
- [ ] No

Fig. 2c, Fig. 2e, Fig. 3d, Fig. 4c, supplement Fig. 44, supplement Fig. 47

Voltage scan conditions

- [x] Yes
- [ ] No

For single-junction solar cells, the scan was performed in the range from 1.3 to 0 V, with a scan step of 20 mV and delay time 40 ms. For tandem solar cells, the scan was performed in the range from 2 to 0 V, with a scan step of 20 mV and delay time of 40 ms.

Explain why this information is not reported/not relevant.

Test environment

- [x] Yes
- [ ] No

The non-encapsulated single-junction and 1 cm2 tandem devices were tested at room temperature and in an N2-filled glovebox. And the encapsulated 64.64 cm2 tandem solar cells were measured in air at room temperature without any temperature and humidity control.

Explain why this information is not reported/not relevant.

Protocol for preconditioning of the device before its characterization

- [ ] Yes
- [x] No

Provide a description of the protocol.

There is no preconditioning.

Stability of the J-V characteristic

- [x] Yes
- [ ] No

Stable power outpot of single-junction and tandem devices were provided in Fig. 2d, Fig. 3e and supplement Fig. 46.

Explain why this information is not reported/not relevant.

3. Hysteresis or any other unusual behaviour

Description of the unusual behaviour observed during the characterization

- [x] Yes
- [ ] No

No obvious hysteresis was observed.

Explain why this information is not reported/not relevant.

Related experimental data

- [x] Yes
- [ ] No

J-V curves obtained from reverse and forward voltage scans were provided in Fig. 3d, Fig. 4d and supplement Fig. 44.

Explain why this information is not reported/not relevant.

4. Efficiency

External quantum efficiency (EQE) or incident photons to current efficiency (IPCE)

☒ Yes ☐ No

EQE curves were provided in supplement Fig. 24 and Fig. 3f.

Explain why this information is not reported/not relevant.

A comparison between the integrated response under the standard reference spectrum and the response measure under the simulator

☒ Yes ☐ No

The integrated Jsc values obtained from EQE were agree well with the Jsc determined from the J-V measurements.

Explain why this information is not reported/not relevant.

For tandem solar cells, the bias illumination and bias voltage used for each subcell

☒ Yes ☐ No

Methods in the supplement text.

Explain why this information is not reported/not relevant.

5. Calibration

Light source and reference cell or sensor used for the characterization

☒ Yes ☐ No

Light source is a Xe arc lamp from an EnliTech solar simulator. The standard solar cells (certified by NREL) were used to check the spectra of solar simulator and EQE equipments.

Explain why this information is not reported/not relevant.

Confirmation that the reference cell was calibrated and certified

☒ Yes ☐ No

The standard solar cell was used as the reference for calibration.

Explain why this information is not reported/not relevant.

Calculation of spectral mismatch between the reference cell and the devices under test

☒ Yes ☐ No

Based on the difference between Jsc and EQE results, the spectral mismatch is negligible.

Explain why this information is not reported/not relevant.

6. Mask/aperture

Size of the mask/aperture used during testing

☒ Yes ☐ No

0.0755 cm2 mask for single-junction solar cells, 1 cm2 and 64.64 cm2 mask for small-area and large-area tandem solar cells, respectively.

Explain why this information is not reported/not relevant.

Variation of the measured short-circuit current density with the mask/aperture area

☐ Yes ☒ No

Report the difference in the short-circuit current density values measured with the mask and aperture area.

Negligible

7. Performance certification

Identity of the independent certification laboratory that confirmed the photovoltaic performance

☒ Yes ☐ No

Large-area solar cells were certified by National Institute of Metrology.

Explain why this information is not reported/not relevant.

A copy of any certificate(s)

☒ Yes ☐ No

Supplement Fig. 45.

Explain why this information is not reported/not relevant.

8. Statistics

Number of solar cells tested

☒ Yes ☐ No

The data from 13 and 20 devices were statistically analyzed for single-junction and small-area tandem solar cells, respectively.

Explain why this information is not reported/not relevant.

Statistical analysis of the device performance

☒ Yes ☐ No

Fig. 2b, Fig. 3b, Fig. 3c, supplement Fig. 19, supplement Fig. 23, supplement Fig. 26.

Explain why this information is not reported/not relevant.

9. Long-term stability analysis

Type of analysis, bias conditions and environmental conditions

☒ Yes ☐ No

Fig. 3h, supplement Fig. 29-39.

Explain why this information is not reported/not relevant.
